# Peptide‐Based Assemblies for Supercapacitor Applications

**DOI:** 10.1002/smsc.202300217

**Published:** 2024-01-24

**Authors:** Sohini Chakraborty, Kamal el Battioui, Tamás Beke-Somfai

**Affiliations:** ^1^ Biomolecular Self-Assembly Research Group Institute of Materials and Environmental Chemistry, Research Centre for Natural Sciences H-1117 Budapest Hungary; ^2^ Hevesy György Ph.D. School of Chemistry Eötvös Loránd University Budapest H-1117 Hungary

**Keywords:** biomimetic materials, charge‐storage mechanisms, peptide assemblies, peptide‐based supercapacitors, secondary structures, supramolecular structures, wearable electronics

## Abstract

The increased focus on green energy storage devices and the related rapid advancement in biomedical technologies makes the investigation of biocompatible integrated systems with medical relevance increasingly important. Peptides and their assembled morphologies with their innate biocompatibility and biodegradability are emerging as promising candidates in this respect due to their structural attributes which can be easily tuned to form supramolecular 3D architectures with extended pathways for ionic mobility. However, to comprehend their applicability in energy storage devices, it is crucial to explore their self‐assembling characteristics, charge‐storage mechanisms, and coating efficacies. Herein, all these aspects are compiled with specific emphasis on peptide‐based systems for supercapacitor applications. The electrochemical charge storage mechanisms that are used for categorizing conventional supercapacitors with the theories and mechanisms outlining biological electron transfer, such as tunneling, hopping, superexchange, and flickering resonance, are collated. Furthermore, the characterization techniques solely pertaining to the study of such systems and their role in predicting the morphology of self‐assembly patterns which could directly impact the overall electrochemical properties are also addressed. Finally, some of the critical challenges associated with these systems while realizing their future potential in the field of sustainable energy storage devices are highlighted.

## Graphical abstract

Peptides and their assembled morphologies are promising candidates for supercapacitor applications due to their structural attributes, which can be easily tuned to form supramolecular 3D architectures with extended pathways for ionic mobility. This review focuses on the various aspects to be considered for the potential usage of these materials at the biotic–abiotic interface, especially in future biomedical implants.

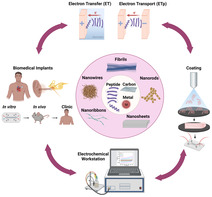

## 1 Introduction

**Figure 1:**
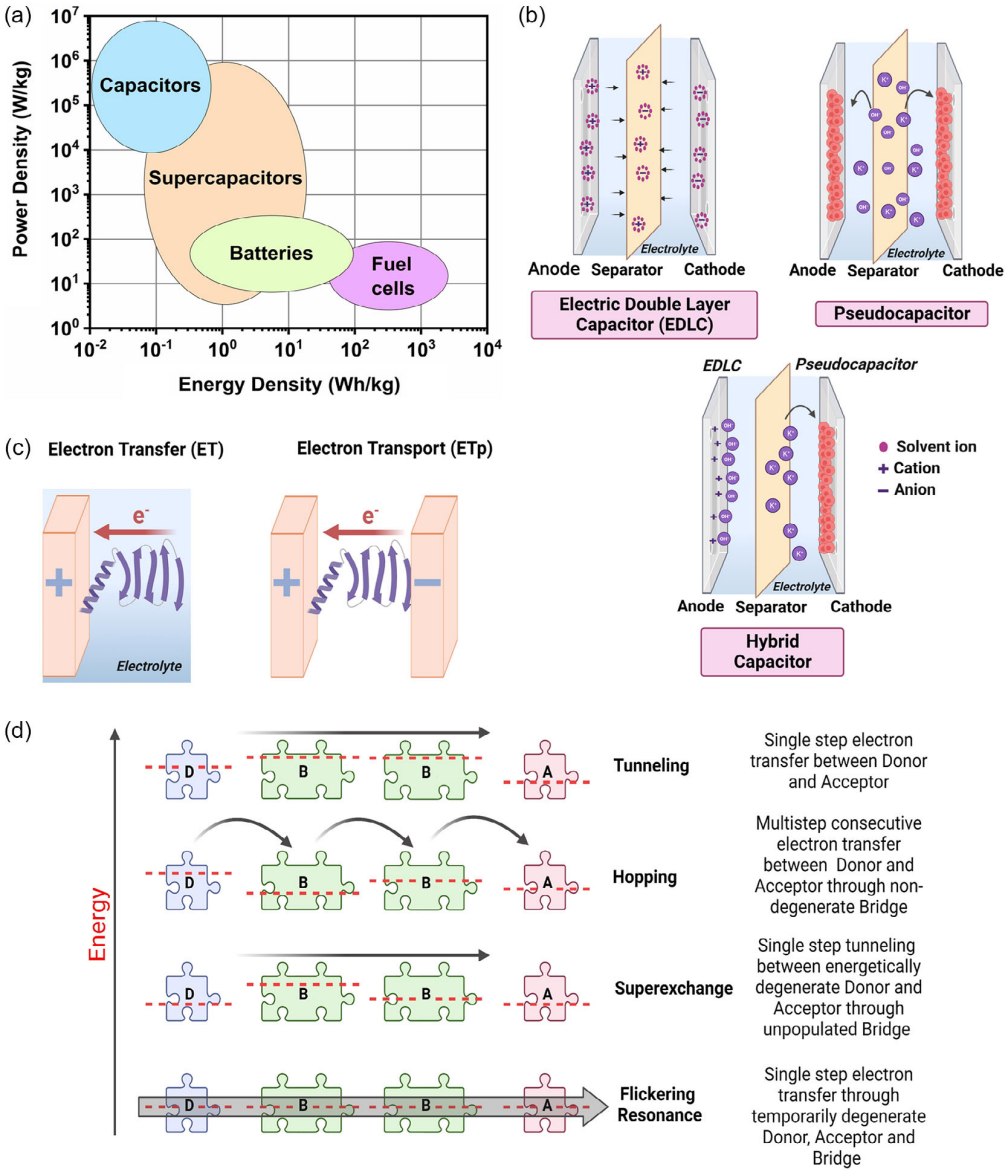
a) Schematic representation of the Ragone plot for various energy storage systems. b) Classification and schematic illustration of the mechanisms of EDLCs, pseudocapacitors, and hybrid capacitors. c) Diagram representing the mechanism of electron conduction in the presence of an electrolyte (ET) and in a solid‐state setup between two electrodes (ETp). d) Energy diagram overview of the various mechanisms of electron conduction, connecting the donor (D) and the acceptor (A) through bridge states (B). The relative energy levels are qualitatively indicated using dotted red lines and the solid black arrows illustrate the direction of the ET process. Figure created with Biorender.com.

Energy is the most potent resource for the effective advancement of our civilization and for the development of innovative technological platforms. With the introduction of more sophisticated systems that ensure a high quality of life, the use of energy has radically increased. This has a direct relation to the alarming pace of climate change, against which rapid and widespread installation of renewable energy sources is a must. At the same time, the fascinating progress in medical sciences demands electronic solutions that could supply modern biotechnological devices. All these directions lead to an increased dependency on sophisticated energy storage and energy harvesting systems. The Sustainable Development Goals embraced by the United Nations General Assembly ensures sustainability through the increased use of renewable sources of energy.^[^ ^1^ ^]^ To realize this goal, many countries have raised their renewable energy targets.^[^ ^2^ ^]^ This would result in an estimated growth in the percentage of renewable energies from 14% to 63% by 2050.^[^ ^3^ ^]^ In alignment with these global policies, to capture various forms of energy and extend its applicability to real‐time devices, the research impetus is focused to a great extent on the development of highly efficient energy storage platforms. Energy can be stored using electrical, mechanical, thermal, and electrochemical energy storage systems. These systems can be differentiated based on the function, time span of storage, rate of response, and type of energy stored.^[^ ^4^ ^]^ Among electrochemical energy storage systems, batteries and supercapacitors (SCs) are the most widely investigated due to their energy and power density characteristics as represented by the Ragone plot (**Figure** **1**a). In this respect, SCs are highly favored as they provide attractive advantages of higher cycling stability and rate capability, high power density, and rapid charge/discharge rates.^[^ ^5^ ^]^ Due to these advantages, they have the potential to complement or substitute batteries in high‐performance electrical machinery. Currently, in most commercial applications, SCs have been used in combination with other energy storage devices such as fuel cells and batteries to maximize energy storage capabilities and charge–discharge characteristics.^[^ ^6^ ^]^ However, extensive research efforts have been dedicated toward the development and commercialization of standalone SC devices with high electrochemical applicability.^[^ ^7^ ^]^

Generally, SCs are classified into electrical double‐layer capacitors (EDLCs), pseudocapacitors, and hybrid capacitors based on their charge‐storage mechanisms (Figure 1b).^[^ ^8^ ^]^ EDLCs store charge electrostatically by the formation of an electrical double layer at the interface of the electrode and the electrolyte. Subsequent diffusion of charges across the separator gives rise to the reversible non‐Faradic storage of energy. In pseudocapacitors, charge storage takes place through Faradic processes via redox reactions on the electrode material. Hybrid capacitors attempt to integrate both electrostatic and electrochemical processes using an EDLC electrode and a pseudocapacitor electrode in a single cell separated by an electrolyte‐soaked membrane. Diverse types of materials have been used to enable charge storage using these mechanisms.^[^ ^9^ ^]^ For example, carbon‐based materials with their high porosity and surface area have been used as EDLC‐type electrodes,^[^ ^10^ ^]^ transition metal oxides with their variable oxidation states,^[^ ^11^ ^]^ and conducting polymers through their doping and dedoping mechanisms have been used as effective pseudocapacitive electrodes.^[^ ^12^ ^]^ Many reviews have outlined the aforementioned conventional mechanisms of SCs.^[^ ^13^, ^14^, ^15^ ^]^ Although each of these types of SCs has distinct shortcomings,^[^ ^13^ ^]^ their applicability often prevails their shortcomings.

With the advancement in technology, the focus has considerably shifted to the development of more functional, biocompatible, miniaturized, and portable energy storage systems that are nontoxic and can be integrated into wearable and clinical electronics.^[^ ^16^ ^]^ This gave rise to the evolution of bioelectronics or bioinspired electronics, which often operates by forming an interface between the abiotic components and biological counterparts of the fabricated devices to enable seamless transduction of electrical signals. Conducting materials that can be used to fabricate these bioelectronics are required to retain their properties and morphology at the tissue interface and possess biorecognition moieties which could effectively assist in cell adhesion.^[^ ^17^ ^]^ These characteristics are highly probable in peptidic assemblies and proteins. Supramolecular structures of these materials displaying conductive characteristics with the help of unique electron transport mechanisms are emerging as materials of interest. Several reports have highlighted the use of carbons derived from silk fibroins, gelatin, chicken egg shell, etc. for SC applications.^[^ ^18^, ^19^, ^20^, ^21^, ^22^ ^]^ DNA‐templated synthesis of metal and metal oxide nanoparticles, semiconductor nanowires, and single‐walled carbon nanotubes has also been reported for electrochemical applications.^[^ ^23^ ^]^ In this regard, peptides are also increasingly considered here, as by now we can effectively engineer them at the molecular level, either into 3D networks or ordered ensembles, which help in providing pathways for ion mobility and tend to expand the surface area available for electrochemical reactions. With more available area, localized reactant concentrations can be avoided and this leads to more efficient electrochemical kinetics and higher areal capacitances.^[^ ^24^ ^]^ Additionally, the submicrometer voids of these architectures can be utilized as scaffolds for the effective incorporation of other conductive additives to obtain a synergistic combination of Faradaic and non‐Faradaic charge‐storage mechanisms which boosts both the power and energy density of the resultant electrode material.^[^ ^25^, ^26^, ^27^ ^]^ Earlier reviews report the importance of nature‐inspired bioelectronics,^[^ ^28^ ^]^ animal‐ and human‐inspired electrode materials,^[^ ^29^ ^]^ protein‐based bioelectronics,^[^ ^30^ ^]^ and sustainable electrochemical devices based on renewable biomolecules.^[^ ^31^, ^32^, ^33^, ^34^, ^35^ ^]^ However, a dedicated overview on the use of peptides and peptide‐based materials for SC applications has not been presented so far. Therefore, we have outlined here the recent advancements in this field, designs and strategies used to develop these electrode materials, their electrochemical performance, and biocompatibility. Further on, the various mechanisms of electron transfer (ET) as well as the challenges faced by such systems and our perspectives for the betterment of these systems have also been included here.

## 2 Electron Conduction in Peptides

With the advances in the field of biologically derived energy storage devices, it has become highly imperative to understand the process of flow of electrons through biological matter which is fundamental in enabling energy harnessing, storage, and utilization at the cellular level. Redox proteins, metalloenzymes, exoelectrogens, or electrochemically active bacteria have been used for the development of bioelectronics.^[^ ^36^, ^37^, ^38^, ^39^, ^40^, ^41^ ^]^ Thus, alongside studying conventional mechanisms of electrical double layer and redox charge transfer, it is crucial to study the electron conduction mechanisms in biological systems. Biological ET is at the heart of processes such as photosynthesis and aerobic as well as extracellular respiration.^[^ ^42^, ^43^, ^44^ ^]^ Several theories, both experimental and computational, have been developed to understand biological ET. The analytical ET theories assume that the pathway of electron conduction is defined by electron‐donating and electron‐accepting cofactors, often connected by a bridge.^[^ ^45^ ^]^ Some of the commonly reported mechanisms for such ETs are tunneling, hopping, superexchange, and flickering resonance.^[^ ^46^, ^47^, ^48^ ^]^ To probe ET through experimental approaches, it is important to realize the wide‐ranging time scales at which these reactions occur, from picoseconds in photosynthetic processes to milliseconds in electron transport involving cytochromes. Nondestructive electrochemical techniques such as cyclic voltammetry (CV) are effective in studying coupling reactions at the submillisecond timescale and have been used as an efficient electroanalytical method to isolate reactions and mechanisms accompanying ET.^[^ ^49^, ^50^ ^]^ Protein films immobilized on suitable electrode substrates have been used to study ET coupling by Armstrong et al.^[^ ^51^ ^]^ They have used optical spectroscopy, pulse radiolysis, nuclear magnetic resonance (NMR), atomic force microscopy (AFM), and scanning tunneling microscopy (STM) to follow the charge transport in proteins. However, ET rates calculated through spectroscopic and electrochemical analysis are numerically distinct as reported by Bostick et al.^[^ ^30^ ^]^ This is because more flexibility with respect to ET pathways and conformations is associated with proteins when using solution‐phase spectroscopic techniques than when using electrochemical techniques where the protein is coated on a solid electrode surface. Thus, with biological systems such as peptides and proteins, the electron conduction mechanisms are highly dependent on the analysis technique and the method of immobilization used.

The flow of electrons through peptides and proteins can be brought about by charge transport within the system and by exchange of electrons with the environment. There are two distinct classifications to categorize the flow of electrons through such systems. 1) ET: movement of electrons through actual difference in the redox potential between an ionically conducting electrolyte and the peptide which is in direct contact with the electrolyte and 2) electron transport (ETp): movement of electrons in the absence of an electrolyte and in the presence of electrodes that are ionically nonconductive (Figure 1c). ETp measurements are carried out in a solid‐state setup by placing the material between two electronically conducting albeit ionically blocking electrodes. In contrast, biomolecular ET processes, also called as redox processes, are analyzed using an electrode (electronically conducting) and a redox electrolyte (ionically conducting).

### 2.1 Electron‐Transfer Mechanisms (ET)

Biological ET often occurs through a series of intermediates rather than in a single step; therefore, the original Marcus theory which described outer sphere ET was extended to include inner sphere ET as well. The electron transport kinetics here can be explained using a first‐order rate constant *k*_ET_ and Marcus theory, as given in Equation (1).^[^ ^52^ ^]^

(1) $$k_{\text{ET}}=\frac{2\pi }{\hbar }H_{\text{AD}}^{2}\frac{1}{\sqrt{4\pi \lambda k_{B}T}}\exp\left(\frac{-{\left(\lambda +\Delta G^{{}^{\circ}}\right)}^{2}}{4\lambda k_{B}T}\right)$$

In Equation (1), −Δ*G* ^o^ is the Gibbs free energy required for the transfer of electrons from the donor to the acceptor, *T* is the temperature, *k*_B_ is the Boltzmann constant, *H*_AD_ is derived from Fermi's golden rule, and it describes the coupling between the electronic state of the donor and the acceptor. *λ* is the reorganization energy required for the rearrangement of the nuclear configuration of the donor and the acceptor to make their energies equal.

The various mechanisms of short‐range and long‐range ET (Figure 1d) were recently reviewed by Ing,^[^ ^53^ ^]^ but due to their close relevance here, we also discuss them briefly below.

#### 2.1.1 Tunneling

Single‐step ET from the donor to the acceptor state is termed as nonadiabatic tunneling. It is a coherent process where the energy of the bridge is higher than that of the donor and thus the electron tunnels across the bridge to reach the acceptor, without residing on it.^[^ ^54^, ^55^ ^]^ Several theoretical models such as the square barrier tunneling model and the bridge‐mediated electron tunneling were refined by Beratan et al. to predict tunneling pathways.^[^ ^56^ ^]^ From the square barrier model, the coupling constant, *H*_AD_, decays exponentially with distance and the decay constant (*β*) is proportional to the effective electron mass and the barrier height.^[^ ^57^ ^]^ Therefore, ET rate is strongly dependent on the distance between the donor and the acceptor. The conductance, in this case, is also exclusively dependent on the distance (R) as depicted in Equation (2).

(2) $$G\propto e^{-\beta R}$$

#### 2.1.2 Hopping

Hopping can be viewed as sequential tunneling between the donor and acceptor states via bridges. It is an incoherent process with the charge momentarily residing in the bridge to give rise to radical species which hops to the acceptor in multiple steps motivated by redox reactions.^[^ ^58^, ^59^ ^]^ The energy of the bridge is lower than the energy of the donor in this case and the charge is injected into the chain when the hole is promoted from the ground electronic state to the excited state above the chain.^[^ ^60^ ^]^ It is more probable for biomolecular systems which have larger distances between their donor and acceptor states.^[^ ^61^, ^62^ ^]^ The rate constant in this case is proportional to the number of steps involved in hopping (*N*) and to the rate of each step involved (*k*_N_), as observed in Equation (3).

(3) $$k_{\text{ET}}\propto k_{N}N^{-\eta }\;\left(\eta \;\text{ranges from}\;1\;\text{to}\;2\right)$$

The conductance in this case is exponentially dependent on temperature. As hopping involves charge localization at each state, appropriate thermal activation is necessary to move to the next one. It follows an Arrhenius temperature dependence and therefore conductance increases with increase in temperature, as given in Equation (4).

(4) $$G\propto e^{-\Delta E_{A}/kT}$$

#### 2.1.3 Superexchange

In this model, the multiple bridge states are instrumental in bringing the energy levels of the donor and the acceptor in resonance, thereby facilitating direct tunneling.^[^ ^63^, ^64^ ^]^ However, it is different from hopping in the fact that the electrons do not reside in the bridge states; rather, the bridge states help in reducing the tunneling barrier and in generating enhanced electronic coupling between the donor and the acceptor states. The donor and the acceptor states are electronically degenerate in this model and the electron easily tunnels from one state to the other through the bridge states. The coupling constant, *H*_AD_, in this case, is distance dependent but it is temperature independent. The proportionality constant *A*, however, is distance independent and temperature dependent as given in Equation (5).

(5) $$k_{\text{ET}}=Ae^{-\beta \left(R-\Delta R\right)}$$

#### 2.1.4 Flickering Resonance

In this mechanism, the donor, acceptor, and the bridge states are made degenerate energetically through thermal fluctuations. This enables the electron to move along the bridge states with almost constant velocity to the acceptor state in order to facilitate coherent tunneling.^[^ ^65^ ^]^ It is different from superexchange in the fact that the bridge states are unoccupied during the ET. Also, it is a coherent process, making it different from hopping. The rate constant depends on an additional probability factor (*P*) which arises from the probability of the multiple states to be energetically degenerate. The ET rate decays exponentially with distance as temperature increases, as depicted in Equation (6).

(6) $$k_{\text{ET}}=\frac{2\pi }{\hbar {\left(4\pi \lambda kT\right)}^{1/2}}V^{2}P$$

Theoretical models are instrumental to analyze ET, and thus related techniques have been used for this purpose, as reviewed by Blumberger et al.^[^ ^66^ ^]^ While we direct the theoretically oriented reader to the in‐depth and high‐quality overview given there, for a brief outlook we summarize here the various computational techniques that can be employed to better understand the aspects of biological electron transport processes. Primarily, theoretical approaches are used to support ET experiments and aid explanation of these preferentially at the atomic level, to predict or explore mechanistic elements that take place on short time scales or design new biological devices and materials, starting by initial computational assessment. Regarding suitability of the computational techniques, for calculating energetic profiles of ET processes, various techniques could be employed, such as, quantum mechanical (QM),^[^ ^67^, ^68^ ^]^ combined quantum mechanics/molecular mechanics (QM/MM),^[^ ^69^, ^70^ ^]^ as well as additional combination with molecular dynamics (MD) methods, for example, the statistics‐based free energy perturbation method (FEP, QM/MM‐FEP),^[^ ^71^ ^]^ which can consider structural dynamics of proteins and peptides in providing free energy values. These techniques are also suitable to identify mechanistic aspects of a specific ET as demonstrated for superexchange,^[^ ^64^ ^]^ tunneling,^[^ ^55^ ^]^ hopping,^[^ ^58^, ^59^ ^]^ and flickering.^[^ ^65^ ^]^ Furthermore, MD‐based approaches can also be employed to understand the propagation effects of ET reactions in the larger spatial area of the surrounding biological environment such as multiheme proteins and DNA where the kinetics of ET have also been evaluated.^[^ ^66^ ^]^

### 2.2 Electron Transport Mechanisms (ETp)

Since, electron transport measurements are performed on systems that are bound to solid metal electrodes, the mechanisms for these processes are less well‐defined. Bostick et al. illustrated that the various ideas and models used for defining ET can be applied to ETp mechanisms as well.^[^ ^30^ ^]^ The donor and the acceptor states have been described as electrodes and the amino acid residues exhibiting varying electrical potential in between them. For a hopping‐like electron transport, the process is incoherent and the conductance depends on temperature similar to that observed in ET. In the case of a superexchange model, the donor and the acceptor states are brought into resonance with the help of the externally applied electrical potential and therefore, the thermal dependences of the donor and acceptor energies are different than that in ET mechanisms. Coherent resonant tunneling and sequential tunneling have also been reported as mechanisms for electron transport and its coherence or incoherence is determined solely by the time spent by the electron on each of the bridge states in between the two electrodes. Flickering resonance has also been used to describe transport across a biomolecule with degenerate donor, acceptor, and bridge levels. Proteins in bioelectronic devices have been assumed as solid‐state conductors and their conductance can be given by the Landauer formula in Equation (7)

(7) $$G=G_{0}T\left(E_{F}\right)$$

where *G*_0_ is the quantum of conductance and *T*(*E*_F_) is the transmission coefficient of the protein as well as the metallic contacts.^[^ ^72^ ^]^

The main challenge in analyzing ET kinetics is associated with the fact that the structure of the peptide or the protein can change when it is immobilized on a solid substrate. Since biological activity is fundamentally dependent on structure, it is highly imperative to immobilize them in a way such as to preserve their biological nature. Immobilization of protein directly on an electrode substrate may suffer disadvantages such as inappropriate positioning of its redox‐active center. In order to enable faster ET kinetics, it is also crucial to have a shorter distance between the electrode surface and the redox‐active center of the protein.^[^ ^73^ ^]^ Several studies report the electron exchange properties of azurin, cytochrome c, fumurate reductase, and other proteins using CV.^[^ ^74^, ^75^, ^76^, ^77^ ^]^ Immobilization can also be carried out through the formation of self‐assembled monolayers via appropriate crosslinking reagents. Silanes, thiols, surfactants, and lipids have been used for this purpose. While silanes and thiols promote peptide self‐assembly on the metal electrode surface, surfactants and lipids create a membrane‐like environment which mimics the natural environment of proteins.^[^ ^78^, ^79^ ^]^

In the case of peptidic systems, the rate of ET is dependent on the peptide backbone, their secondary structure, the nature of amino acids, and length of the sequence.^[^ ^80^ ^]^ The helical backbone has been extensively studied for their ET mechanisms as they are predominant in biological electron‐transfer systems.^[^ ^81^, ^82^ ^]^ In several notable cases, α‐aminoisobutyric acid (Aib) has been used as a component amino acid to promote the formation of helical motifs.^[^ ^83^, ^84^ ^]^ Yu et al. studied the mechanism of ET for Aib‐rich peptides and concluded that on increasing the conformational rigidity of the backbone, the ET rate reduces as additional energy is required to align the backbone torsional motion to enable easy intramolecular ET along the peptide backbone.^[^ ^85^ ^]^ This study was further extended to include alkene‐rich side chains in the peptide backbone.^[^ ^86^ ^]^ Improved ET efficiencies were observed by fine tuning the conformational rigidity of the backbone and incorporating alkenes as the electron‐rich centers. Additionally, the type of helix also played an important role in affecting the rate of ET. The photocurrent efficiency for the α‐helix (3.6_13_‐helix) conformation of the peptide was observed to be greater than the 3_10_‐helix by Gatto et al.^[^ ^87^, ^88^ ^]^ This arises from the orientation of the dipoles, resulting from the position of the amino acids. The conformation could also be switched between α‐helix state and 3_10_‐helix for helical dodecapeptides as a result of the interaction between the dipole moment and the external electric field.^[^ ^89^ ^]^ Therefore, for these systems, it is crucial to achieve specific structural attributes that could positively impact ET efficiencies. After immobilization, the surface properties of these systems can be evaluated through techniques such as circular dichroism (CD) spectroscopy, UV–vis spectroscopy, quartz crystal microbalance, STM, AFM, etc. The responses obtained from various electrochemical techniques coupled with the understanding of the structure, thickness, orientation, and stability of the immobilized material help in providing a detailed picture of the plausible mechanisms underlying efficient electron transport.

## 3 Factors Responsible for Electron Transfer in Peptides

There are various factors which contribute to ET in peptides and these are decisive in determining the type of mechanism adopted for electronic conduction as reviewed by Shah et al.^[^ ^90^ ^]^

### 3.1 Presence of Redox‐Active Centers

Metals in metalloproteins or peptide complexes can act as redox active centers by promoting electron transport through redox processes. In this case, hopping would be the more preferred mechanism of electron conduction due to the presence of redox active sites which can act as junctions for the relay of charges and subsequent electron conduction.^[^ ^82^, ^91^, ^92^ ^]^

### 3.2 Peptide Chain Length

As mentioned earlier, tunneling depends strongly on the distance between the donor and the acceptor states. Therefore, as the distance increases, there is a switch from tunneling to the hopping mechanism for peptidic systems.^[^ ^86^, ^93^, ^94^ ^]^

### 3.3 Presence of Side Groups and Pendant Groups

Aromatic residues in peptides initiate π–π stacking which aids in lowering the bandgap.^[^ ^52^ ^]^ Electron hopping is plausible in this case due to the formation of delocalized bands or aromatic bridge states. Additionally, protonation of amino groups can increase or decrease the ET efficiency depending on the influence of the Coulombic energy and the activation energy as per the Marcus theory.^[^ ^95^ ^]^

### 3.4 Secondary Structures

ET is highly dependent on the dipole moment of the peptide from the C‐ to N‐terminus.^[^ ^96^ ^]^ For α‐helices, the change in dipole is more significant per residue when compared to β‐strands.^[^ ^97^ ^]^ This enhances the transfer to a great extent for an α‐helical peptide.

## 4 Factors Influencing Peptide Assemblies

**Figure 2:**
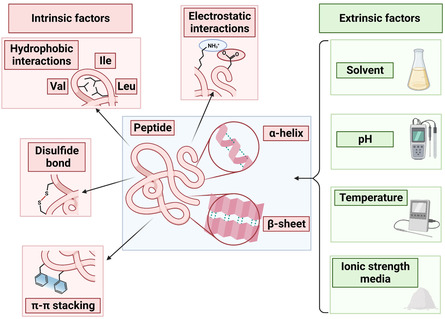
Schematic representation of the intrinsic and extrinsic factors affecting formation of nanostructures via self‐assembly. The α‐helix and the interstrands of the *β*‐sheet conformation are stabilized by hydrogen bonds as depicted by dashed green lines. Figure created with Biorender.com.

The morphology of any peptide‐based supramolecular network as a result of self‐assembly has a decisive effect on its conductive properties. The interactions responsible for the effective formation of peptide assemblies are typically secondary interactions, such as π–π interactions, hydrogen bonding, hydrophobic, and electrostatic effects (**Figure** **2**). By changing the hydrophilicity or the hydrophobicity of the peptide chains, the morphologies can be varied from vesicles to tubes and ribbons.^[^ ^32^ ^]^ Tubular structures have been reported to exhibit enhanced conductivity due to the presence of interconnected nanonetworks all throughout the structure.^[^ ^98^ ^]^ Self‐assembly formation through the stacking of aromatic rings is driven thermodynamically by the energy contributed from this process.^[^ ^99^ ^]^ π–π stacking patterns also orient the assembly process in a specific direction and promote directional growth, leading to the formation of more ordered structures. The propensity of assembly formation in peptides is also governed by the presence of ample hydrogen bond‐forming sites. The morphology of peptide assemblies can be easily manipulated by restricting the formation of hydrogen bonds at specific locations. This can be carried out by performing selective N‐methylation of amino acids in the sequence to orient self‐assembly toward a desired nanostructured morphology.^[^ ^100^ ^]^ Electrostatic interactions also play a major role in the formation of higher‐order peptidic supramolecules, especially for sequences containing positively or negatively charged amino acids. The charge of the related side chain groups is typically dependent on pH and ionic strength and therefore can be regulated easily by altering these parameters. In addition to these interactions, assemblies can also be formed by taking advantages of specific bond affinities. For instance, formation of disulphide and imine bonds has also been instrumental in forming self‐assembled states through covalent interactions.^[^ ^101^ ^]^ Furthermore, the conductance of peptide nanostructures was also found to be dependent on the peptide folding state and the humidity content.^[^ ^102^ ^]^ By altering the humidity conditions, the type of charge carrier responsible for charge transport was identified and fully folded peptide fibrils demonstrated higher conductivity signifying the importance of secondary structures on the mechanism of conduction.

Other intrinsic factors such as the hydrophobicity of the amino acid of the side chains and their arrangement in the peptide sequence also impact the formation of peptidic assemblies. For example, diphenylalanine (Phe‐Phe) self‐assembles into nanotubes while diphenylglycine (Phg‐Phg) forms nanospheres though their properties are similar.^[^ ^103^ ^]^ This is because the side chain of phenylalanine is more flexible due to the presence of a methylene group.^[^ ^104^ ^]^ Besides the individual amino acids as described above, peptide assemblies themselves are also affected by external factors such as pH, temperature, and the solvent used. Additionally, Miravet et al. reported that the morphology changes from nanotapes to micelles as the temperature increases.^[^ ^105^ ^]^ In a similar study by Hamley et al. it was also observed that the peptide C_16_‐KKFFVLK with an amphiphile linker could either form nanotubes or helical ribbons upon self‐assembling at room temperature.^[^ ^106^ ^]^ After heating up to 328 K, the macroscopic shape changed to twisted tapes which reverted back to the original morphology once cooled to room temperature. Thermal conductivities and hydrophobicity index of the substrate also affect the extent of self‐assembly. In the case of Phe–Phe (FF) nanotubes, higher density of self‐assembled tubes was found on polyvinyl chloride (PVC) substrate than on aluminum and mica substrates whereas the morphology changed entirely on glass and silicon substrates.^[^ ^107^ ^]^ This was mainly attributed to the hydrophobic nature of FF, as a result of the aromatic rings in its structure. Since PVC substrate has the highest hydrophobicity, it coats better on it when compared to other substrates. Additionally, the thermal conductivity of the substrate also influences this phenomenon. Substrates with lower thermal conductivity such as PVC slow down the evaporation of the solvent, allowing ample time for the nucleation to occur, leading to the formation of nanotubes with the help of hydrogen bonding interactions. Varying pH also results in differences in the morphology of self‐assembled structures. When the peptide EAK‐16 (Ac‐AEAEAKAKAEAEAKAK‐NH_2_) was subjected to different pH conditions, it exhibited a fibrillar structure at pH 4 which changed to a globular morphology at pH 6.8 and then it reverted back to the fibrillar structure at pH 11.^[^ ^108^ ^]^ At lower pH, the glutamic acids get protonated, whereas at much higher pH, lysines get deprotonated. Therefore, at these pH values, the electrostatic interactions are reduced, which result in nanofiber formation. The effect of solvent on the morphology of diphenylalanine has been investigated by Huang et al.^[^ ^109^ ^]^ Diphenylalanine in water forms hollow tube‐like structures with diameters ranging a few micrometers. On adding around 10% of acetonitrile, a mixture of tubes and fibers was formed and in 100% acetonitrile, only nanofibers are formed. This solvent‐triggered shift in morphology from microtubes to uniform nanofibers has been anticipated to find applications in energy storage devices and biosensing platforms.

## 5 Coating Techniques

**Figure 3:**
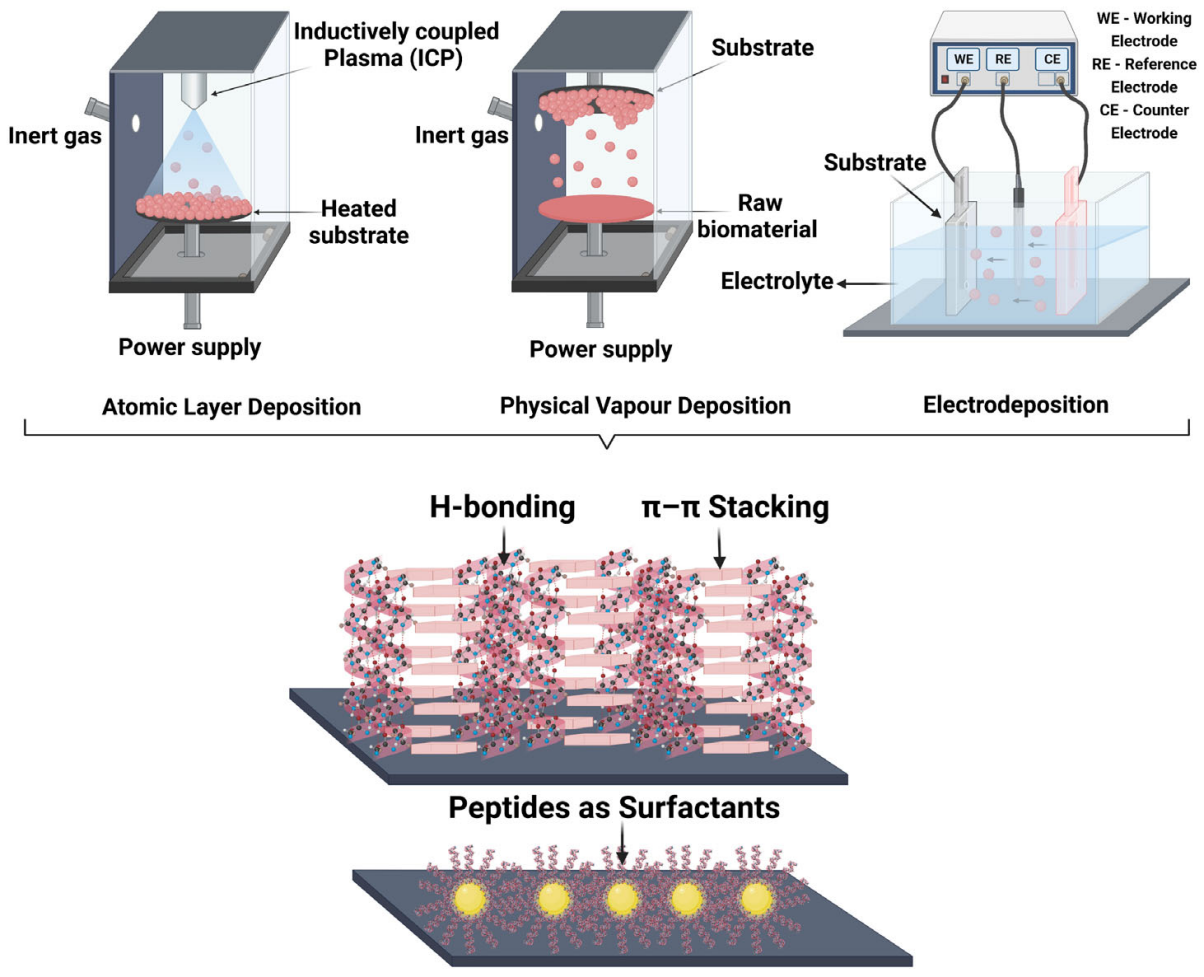
Schematic overview on the deposition techniques used for peptidic systems on electrode substrates. The basic principles for the three different deposition techniques are depicted followed by the various interactions which give rise to specific morphologies. For instance, sequential π–π stacking and hydrogen bonding interactions can give rise to a nanosheet‐like morphology while a nanosphere‐like morphology is more probable, when the peptides act as surfactants. Figure created with Biorender.com.

For the effective usage of peptides as electrode materials, it is imperative to identify efficient coating techniques which retain their original structure and self‐assembling attributes, thereby enabling ET. Naturally, existing coating techniques have found their way into this area; however, several of them need modifications in order to be effectively adopted for peptide systems (**Figure** **3**). Here, we highlight some of the main techniques used for coating peptides on electrode substrates and also focus on the alterations in setups or protocols that enable their application for peptidic compounds.

### 5.1 Physical Vapor Deposition (PVD)

Physical vapor deposition (PVD) is a method of forming thin‐film coatings by vaporizing a solid material in vacuum for subsequent deposition on a suitable substrate. It has been widely used in the development of microelectronics as it forms films in the micrometer/nanometer range and can also be used for multilayer coatings. Rosenman et al. suggested the use of PVD as a technique to form vertically aligned peptide nanotubes.^[^ ^110^ ^]^ Traditional PVD employed high temperature and vacuum conditions. For peptidic systems, high temperature can induce structural transformation which can obstruct the assembly formation. Therefore, the biomolecule deposition system developed by Rosenman and co‐workers emphasized specifically on the complexity of such materials. The setup consisted of a vacuum chamber with a sample holder, a substrate holder, and a piezoelectric quartz unit for controlling the thickness of the deposited film. The temperature of the substrate and the sample were monitored by externally fitted control systems. The temperature range of sublimation for the peptide to be deposited was used for setting the temperature of the control system. The temperature ranges available for the substrate and the precursor are 25–150 and 25–300 °C, respectively. Additionally, a vacuum pressure of up to 2 × 10^−6^ mbar could be supplied by the pump in this setup. The peptide nanotubes and nanofibers grown by this technique showed a distinct quantum confinement effect, which gave rise to remarkable photoluminescence and thus could be used in bioinspired light‐emitting devices.^[^ ^111^ ^]^ Moreover, the peptides deposited on carbon electrodes for SC applications also exhibited a remarkable capacitance enhancement.^[^ ^112^ ^]^

### 5.2 Electrodeposition

The organization of organic and inorganic domains to affect the formation of hybrid nanostructures is significant from the perspective of device fabrication.^[^ ^113^ ^]^ Electrochemical deposition or electrodeposition is an inexpensive and facile technique which uses electric current for the reduction of metal ions to fabricate ordered nanostructured films. The structural aspects of the deposited films can be regulated by altering the electrochemical parameters such as the potential window, concentration of the solutions, and the temperature.^[^ ^114^ ^]^ Additionally, electrodeposition also ensures tighter packing efficiency and uniformity of the resulting films.^[^ ^115^ ^]^ In this respect, peptides have been used as surfactants for the effective growth of organic–inorganic hybrid structures by electrodeposition.^[^ ^116^ ^]^ Amphiphilic peptides with self‐assembling characteristics can serve as alternatives to traditional surfactants such as sodium dodecyl sulfate (SDS) and cetyltrimethylammonium bromide (CTAB). They are capable of interacting with the inorganic counterpart at the interface of the working electrode during electrodeposition due to the electrostatic interactions between the charges at the surface and the carboxyl functional group. They also tend to self‐assemble as a result of the interfacial tension at the electrode–electrolyte interface and act as effective capping agents to prevent the aggregation of the inorganic nanoparticles.^[^ ^117^, ^118^ ^]^ Peptide surfactants have been used for various applications such as drug and gene delivery, tissue engineering, and as a template for nanofabrication.^[^ ^119^ ^]^ Utilizing this property, electrodeposited nanohybrid films of peptides have been leveraged for their electrochemical and optoelectronic properties and as a coating to enhance cell adhesion of titanium based implants.^[^ ^120^ ^]^ Electrodeposition of peptide/metal hydroxide hybrids have been performed wherein the peptides act as surfactants for the electrochemical growth of unique nanostructures.^[^ ^121^ ^]^ In a similar study, the in situ fabrication of a dipeptide/cobalt hydroxide nanocomposite was developed as electrode materials for SCs.^[^ ^122^ ^]^

### 5.3 Atomic Layer Deposition (ALD)

Atomic layer deposition (ALD) represents a relatively newer approach toward the surface engineering of electrodes for energy storage devices. Its main advantages include the ability to form conformal coatings of composite structures at low growth temperatures with uniform thickness, enabling atomic‐scale stoichiometric deposition.^[^ ^123^ ^]^ It is a subcategory of chemical vapor deposition and involves the adsorption of the precursor on the exposed surface of the substrate. The process is self‐limiting and the adsorption stops once the available sites are used up ensuring the formation of thin films. There are two types of ALD, namely, thermal enhanced and plasma enhanced ALD. Plasma‐enhanced ALD is a method which can be used for low‐temperature applications. This approach can be particularly useful for peptidic systems as they are sensitive to higher temperatures. Thin films deposited at the electrode/electrolyte interface enhance ionic diffusion and also aid in safeguarding against the structural distortions and volumetric changes that may occur during the charge/discharge process. It is being widely used in industries for device fabrication, as thin films and coatings can contribute greatly to miniaturizing electronics. Complex 2D and 3D nanostructures can also be deposited as thin films efficiently using this technique. ALD has been used to create a 3D network of nanoribbons using TiO_2_ deposited on a peptide template.^[^ ^124^ ^]^ The formation of the 3D network was used as an electrode material for lithium secondary batteries. It exhibited high specific capacity and rate capability along with enhanced cycling stability. Interestingly, the system also exhibited high thermal stability that is uncharacteristic to most biomolecules and largely retained its nanoscale structure during the vacuum deposition process. Additionally, the developed 3D framework showed UV‐switchable wetting properties.^[^ ^125^ ^]^ Wearable SC electrodes have also been fabricated using peptide‐Co_9_S_8_ nanobricks synthesized using the ALD technique.^[^ ^126^ ^]^

### 5.4 Other Coating Techniques

**Figure 4:**
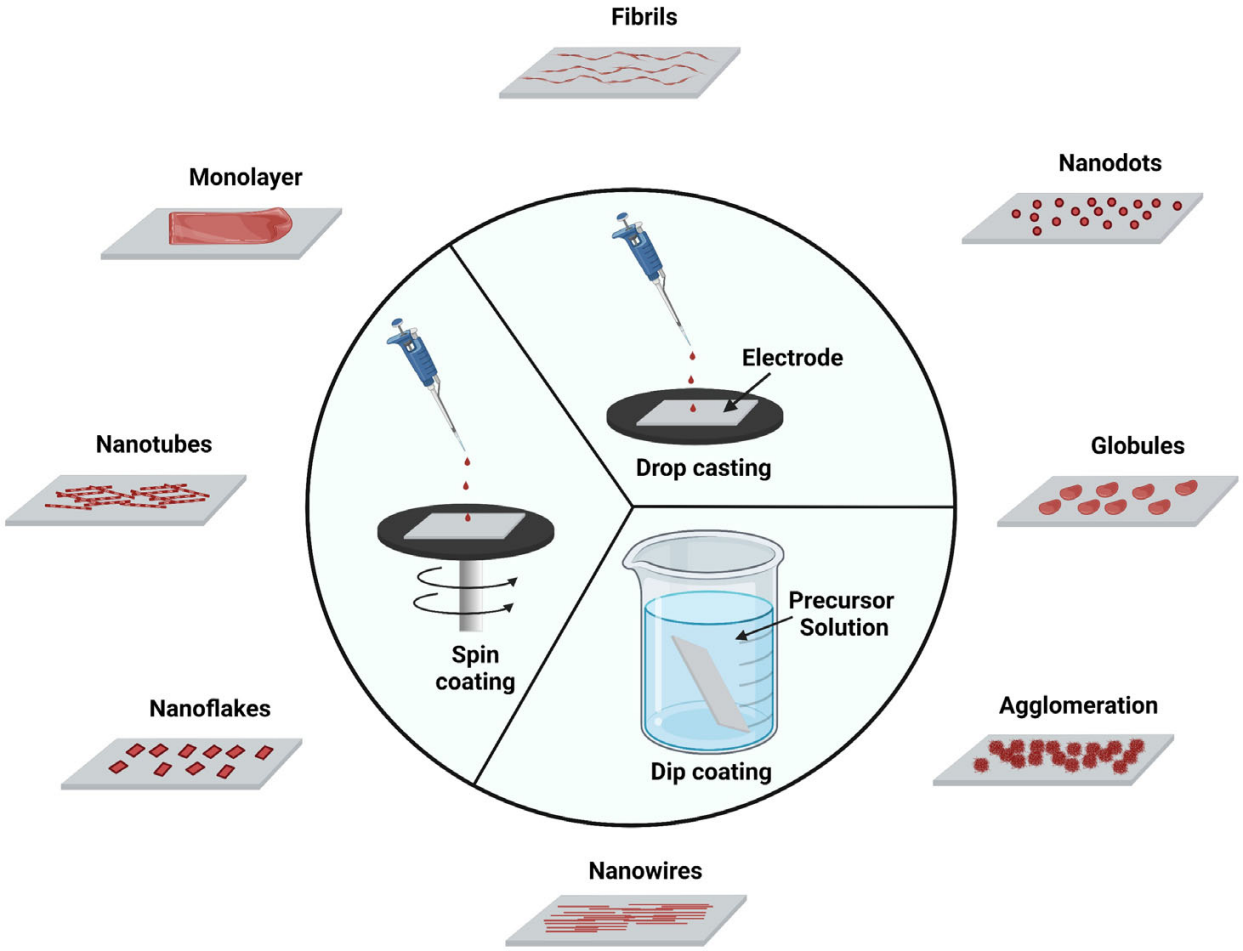
Overview diagram representing the spin‐coating, drop‐casting, and dip‐coating techniques. The use of different coating techniques gives rise to different morphologies for peptidic systems. The most commonly observed morphologies are depicted. Figure created with Biorender.com.

The immobilization of peptides and proteins on the surface of the substrate can also be performed through drop casting, spin coating, and dip coating (**Figure** **4**). In drop casting, small volumes of precursor solutions are dropped onto the surface of the substrate and allowed to dry in nitrogen atmosphere to prevent oxidation or other side reactions. The process is repeated many times to obtain a uniform deposition. It is easy to use; however, it suffers from disadvantages such as the inability to control the thickness and uniformity of coating. Dip coating involves dipping the electrode into the precursor solution for a predefined time period and then drying it in nitrogen atmosphere to obtain the coated electrode. Many dipping cycles are carried out to ensure efficient coating. This process enables higher reproducibility of electrochemical properties, more control over thickness, uniformity of the films, and greater surface area coverage. However, it requires more material and it is a time‐consuming process. In case of spin coating, the precursor solution is dropped in the center of the substrate and then spun at a high speed to obtain a thin film. This method is faster, cost‐effective, and ensures higher reproducibility. Wasilewski et al. reported the comparison of the biosensing properties of peptides coated using three immobilization techniques, drop casting, spin coating, and dip coating.^[^ ^127^ ^]^ Based on this systematic comparison, the highest efficiency was recorded for the dip‐coated peptide‐based sensor. Another study showed that the morphology of the self‐assembled peptides can be modulated by varying the parameters during the spin‐coating process.^[^ ^128^ ^]^ Four peptide analogues, FF, Boc‐FF, FF‐OMe, and Boc‐FF‐OMe, were used and their morphologies were evaluated through drop‐casting and spin‐coating techniques. For Boc‐FF‐OMe, at lower spin rates, the peptides formed short fibrils. On increasing the spin rate, it formed isolated peptide globules. On drop casting, the peptide exhibited randomly oriented long fibrillar morphology. Similarly, FF shows fibrils and a flake‐like morphology, using drop‐casting and spin‐coating methods, respectively. For Boc‐FF, both the methods show the formation of globules, which are more closely packed in the case of drop casting. On the other hand, FF‐OMe forms long straight fibers when drop cast in contrast to bigger drop‐like morphology when spin coated. The differences in morphology on spin coating and drop casting were attributed mainly to the fact that the deposition profile in the latter is characterized by peripheral agglomeration which disturbs the long‐range order. However, spin coating is associated with high rotational velocity which helps in forming more uniform films. This aspect has also been explored as an alternative method for the directed self‐assembly of amyloid Aβ peptides by Fakhraai et al.^[^ ^129^, ^130^ ^]^

## 6 Techniques for Studying Biomolecules in Energy Storage Devices

The role of peptides in bioelectronics can be realized more effectively by understanding the natures and types of supramolecular structures formed as a result of self‐assembly. Simple spectroscopic techniques such as UV–vis, IR, fluorescence, NMR, and CD spectroscopies can predict relevant information about the local secondary structure, the spatial 3D arrangement, or the tertiary structure of the peptides and small proteins. When complemented with imaging techniques such as transmission electron microscopy (TEM) and AFM, they can jointly provide insight into the properties of these complex assemblies. Peptide conformational changes can be effectively captured through CD spectroscopy. The n‐π* and π–π* electronic transitions are observed as positive and negative bands in the CD spectra which can be used to predict the secondary structure of peptides. IR spectroscopy can be employed in addition to CD spectroscopy to identify the secondary structures based on the IR signals in the amide I region. The amide bonds of peptides can also be detected in the far‐UV region between 180 to 230 nm and the near‐UV region from 240 to 295 nm, which is characterized by peptides containing aromatic residues.^[^ ^131^ ^]^ Additionally, oxidation reactions could be detected by identifying specific peaks in the UV spectrum. A blueshift in peak positions is also observed in case of polar solvents whereas the second derivative of the UV spectra reports conformational changes arising as a result of self‐assembly and supramolecule formation. The formed macroscopic assemblies often share properties with fibrillary morphologies, and such formation of amyloid‐like constructs can also be tracked using, for example, Congo Red as an extrinsic probe.^[^ ^132^ ^]^ On the addition of Congo Red, a redshift is observed in the spectra accompanied by an increase in light absorption. β‐sheet formation can also be detected using fluorescent probes such as Thioflavin‐T and Congo Red, which promote an increase in fluorescence intensity as a result of binding between the dye and the peptide.^[^ ^133^ ^]^ The chemical shifts obtained from NMR spectroscopy give information on the secondary structure, interatomic distance between residues, and the 3D arrangement of atoms.^[^ ^134^, ^135^ ^]^ MD simulations with the help of force fields containing the parameters of the specific compounds can also be used to interpret structural parameters obtained from such experiments to arrive at plausible molecular structures.^[^ ^136^, ^137^ ^]^ They present a theoretical point of view, where electronic properties and performance can be tested prior to experimental efforts aiming to create such systems.

## 7 Peptide‐Based Supercapacitors

**Figure 5:**
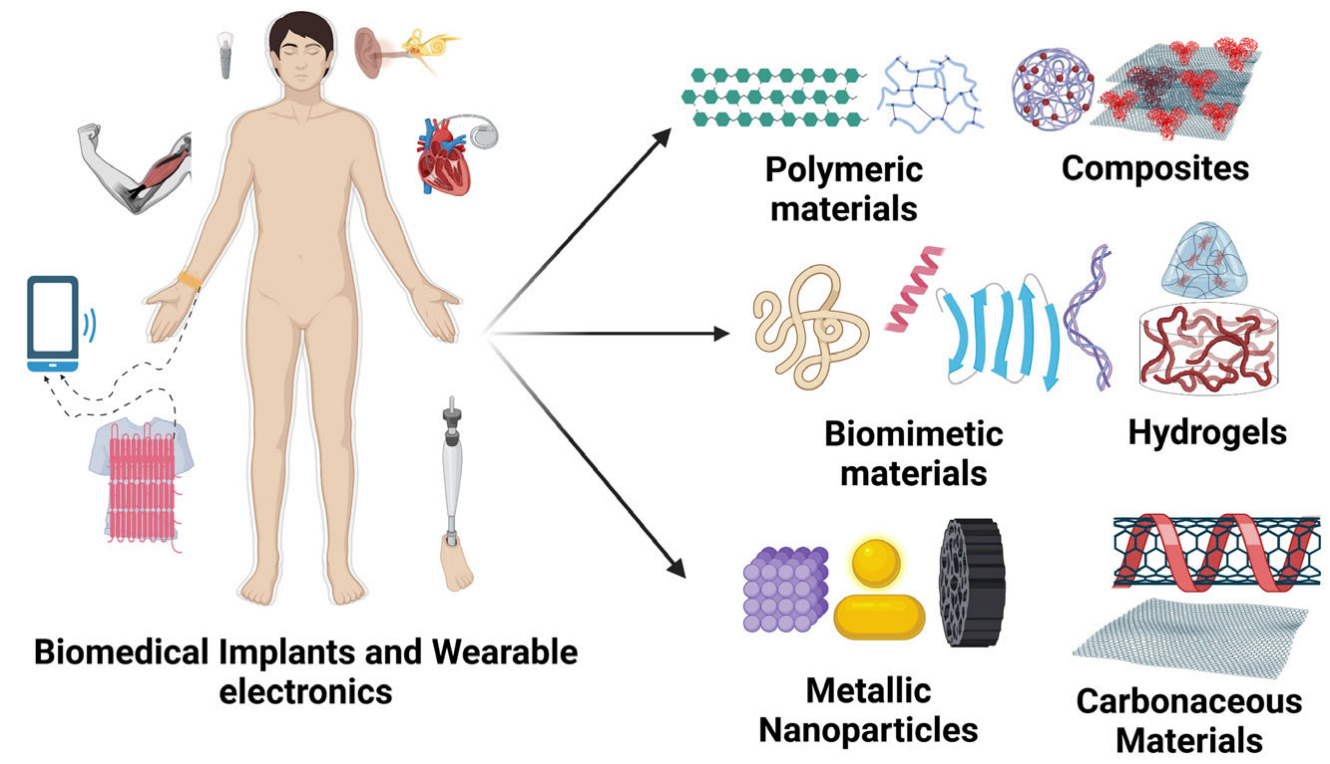
Schematic representation of various materials used for the fabrication of biomedical implants and wearable electronics. Figure created with Biorender.com.

Conventional electronics rely heavily on the use of inorganic materials. However, to develop more futuristic high‐performance implantable and wearable electronics, several related areas and material properties need to be further developed. Among others, soft organic materials with higher mechanical flexibility, larger surface area, low processing temperatures, rapid ion transport, flexibility to chemical modifications, regulated biological properties with adaptable biorecognition elements, and higher conductivity are all requirements that need to be addressed in the future.^[^ ^138^ ^]^ To this end, conducting polymers have been used to enhance functionality at the biotic–abiotic interfaces. Invasive devices such as biosensors, pacemakers, biomedical devices, cochlear implants, etc.^[^ ^139^ ^]^ and also noninvasive wearable devices have been efficiently developed using these polymers.^[^ ^140^ ^]^ However, hard electronic materials used in implantable devices often generate a foreign body response (FBR), which could lead to inflammatory reactions.^[^ ^138^ ^]^ Biomimetic materials such as peptide‐based systems (**Figure** **5**) can be used to mitigate this response and enable facile integration in biomedical implants as they offer the flexibility to design versatile assemblies with relevant properties.

Based on their specific attributes, we have divided the discussion of peptide‐based SCs into three subsections. While 7.1 focuses on the systems where peptides and their self‐assembly is the key for reaching the final constructs, Section 7.2 and 7.3 detail those where the peptides are combined with carbon and metal‐containing systems, respectively.

### 7.1 Peptide Assemblies

The vast field of functional SCs are majorly governed by carbonaceous materials. One of the major challenges in this field is the development of more biocompatible and biodegradable SC devices. Fitting into this direction, after demonstrated nanotube forming capacity of self‐assembling peptides,^[^ ^141^ ^]^ that was explored by us theoretically even for non‐natural peptides,^[^ ^142^, ^143^, ^144^, ^145^ ^]^ peptide nanotubes (PNT) have been investigated by Beker et al.^[^ ^111^ ^]^ In contrast to carbon nanotubes as SC electrodes, they have been reported to exhibit large EDLC. The morphology‐driven effect on supercapacitive properties was demonstrated. Hollow PNTs composed of linear diphenylalanine peptide (FF) peptide molecules enhanced the double‐layer capacitances. On the contrary, fiber‐like PNTs obtained from cyclic peptides which were hydrophobic in nature did not display any supercapacitive behavior. The lyophilized FF peptide was coated on a porous carbon electrode by the PVD technique. The coating technique played a vital role for the effective fabrication of SC electrodes. Factors such as uniformity of the coated material and high adhesion to the substrate are extremely important to obtain high capacitances. Rapid evaporation using hexafluro‐2‐propanol as the solvent resulted in a nonhomogeneous coating with low adhesion to the substrate. To avoid this issue, PVD was used as the coating technique to deposit homogenous, thick, and aligned nanoarrays of PNTs on the carbon working electrode. 1 m H_2_SO_4_ was used as the electrolyte, carbon electrodes were used as the counter electrode, and Ag/AgCl was used as the reference electrode. The PNT was found to self‐assemble into two distinct morphologies, closed‐end and open‐end nanotubes. At higher temperatures, however, the morphology changed significantly leading to the formation of fiber‐like PNTs. This was mainly attributed to the cyclization of the linear peptides during the PVD process. The specific capacitance from the cyclic voltammogram was found to be around 80 F g^−1^ which was due to the large functional surface area and the wettability of the fabricated PNT. The open‐end nanotubes were held responsible for the contribution to the enhanced supercapacitance due to the presence of nanoscale hydrophilic channels which allowed the easy movement of aqueous electrolytes through those.

This work was further extended to include the effect of different height and density of the PNT coatings on the obtained double‐layer capacitances.^[^ ^112^ ^]^ The effect of thickness was evaluated using 10 and 40 μm of the PNT coating to obtain specific capacitances of 160 and 240 μF cm^−2^ respectively. This indicated that wettability of the electrode by an electrolyte was a key parameter to enable high specific capacitances. It also helped in reaffirming the presence of hydrophilic channels in the structure of PNT which increased the wettability and gave rise to enhanced capacitances.

Further investigations on the influence of the conformation of peptides and their subsequent formation into self‐assembled nanostructures for biocompatible SC applications have been reported by Hu et al.^[^ ^146^ ^]^ An in‐tether chiral center was used to strategically induce the formation of a helical peptide. By substituting phenyl groups and naphthyl ones, ample intermolecular π–π* interactions were introduced which accelerated the process of self‐assembly to a great extent. The cyclic pentapeptide used for the studies had the common sequence of Ac‐CAAAS5(X)‐NH_2_ where X represents methyl, phenyl, or naphthyl substituents. Six peptides were obtained by synthesizing these sequences in both R and S configurations and all the R‐configured peptides were found to be helical in nature. The latter secondary structure and the presence of aromatic substituents promoted self‐assembly. The mechanism of self‐assembly was proposed to be diffusion‐limited crystal growth which could explain the presence of both nanobelts and nanotubes. Using density functional theory (DFT) calculations, the authors identified that the peptide chains possessed a polar backbone and a nonpolar side chain with a zipper‐like superhelical assembly. On extending this approach to other sequences, comparable results were obtained with different kinds of nanostructures. This established that the design of the nanostructures obtained was dependent on the sequence used. By exploiting this advantage, the applicability of these peptides in biocompatible SC electrodes was evaluated. The electrochemical studies using modified glassy carbon electrodes as the working electrode and Hg/HgO as the reference electrode showed typical capacitive characteristics and enabled a maximum capacitance retention of 80% after 5000 cycles. The energy storage capacity was also found to vary with the difference in the morphology and the sequence used. The capacitance values also increased upon using 0.1 m H_2_SO_4_ as electrolyte instead of 0.05 m KH_2_PO_4_/0.5 m KCl. A stable capacitance of 2.35 mF cm^−2^ was obtained at the current density of 50 μA cm^−2^.

**Figure 6:**
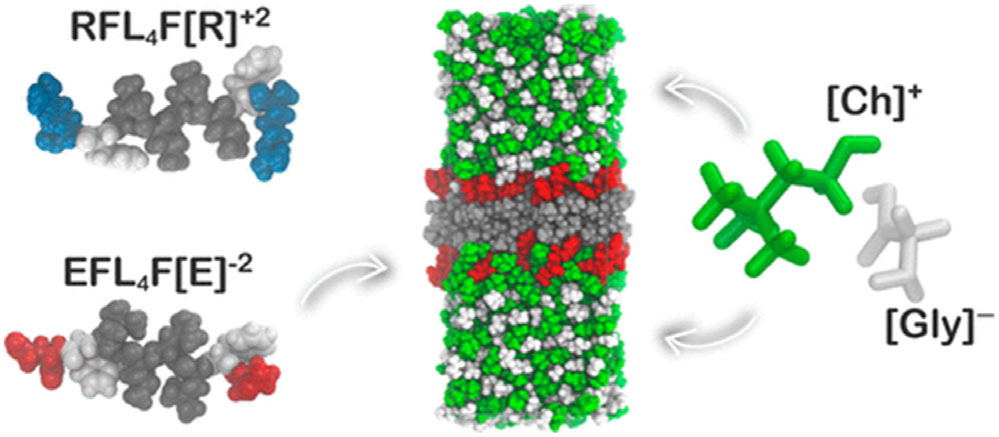
Right: Molecular representation of the bolaamphiphilic polypeptides and their net charge. Left: The ionic pair that constitutes the SC electrolyte. Middle: The computational cell showing the stabilized structure of the nanosheet (in this case specifically the EFL_4_FE) in the ionic liquid [Ch][Gly]. The hydrophobic core of the nanosheet is represented in gray, the charged groups in red, and the ionic pairs in white and green. It is possible to observe a slight permeation of the cations on the negatively charged surface of the nanosheet. Analogous representation can be obtained for the RFL_4_FR system. Reproduced with permission.^[^ ^137^ ^]^ Copyright 2018, American Chemical Society.

The energy storage capability of these peptides has also been studied from the computational point of view through atomistic stimulations to give a better understanding of the internal redox processes. Colherinhas et al. proposed a completely biodegradable SC system using peptides as electrode materials and an amino acid‐based ionic liquid as electrolyte.^[^ ^137^ ^]^ The selected systems were studied using MD simulations using the recent CHARMM36^[^ ^147^ ^]^ force field suitable for biomolecular simulations and the GROMACS 2016^[^ ^148^ ^]^ software package that is one of the commonly used programs for biomolecular studies. Bolaamphiphilic peptides with two hydrophilic ends separated by a hydrophobic core have been reported to demonstrate high stability and thus they have been selected as functional materials for the SC electrodes. The design of the peptide nanosheets was chosen to be such as to effect greater mechanical and structural stability along with high density of surface charges. The peptides chosen were RFL_4_FR and EFL_4_FE wherein leucine (L) functioned as the hydrophobic separator between the FR and FE terminals (**Figure** **6**). The terminals FR and FE were also strategically chosen to impart π–π interactions due to phenylalanine (F) and the polar residues. The arginines (R) and the glutamic acids (E) improved the wettability of the peptide‐based electrodes with the electrolyte. An ionic liquid using cholinium cation and glycine as the anion was used as a nontoxic and biodegradable electrolyte. The assembled SC acts as a double‐layer electrical capacitor due to the hydrogen bonded interactions at the electrode–electrolyte interface. The contribution to specific capacitance was obtained from the heteroatoms in the carbonyl oxygen and the amino groups of the peptides, which rendered pseudocapacitance to the system, in addition to the acquired asymmetry as a result of the interactions at the negative and positive electrodes. The applied potential difference was well within the conventional window of ionic liquids and the specific capacitance was found to be 18.7 μF cm^−2^, which is much higher than that for graphene‐based SCs in ionic liquid electrolytes. The individual electrode capacitances of the cathode and the anode further reiterated the asymmetry of the SC device. To compare the results obtained, simulations were conducted using the same ionic liquid electrolyte and gold electrodes. The capacitance values were seven times lower than that achieved for the polypeptide.

Similarly, Alves et al. recently demonstrated A_6_R heptapeptide models using MD simulations where the temperature dependence of various parameters, such as interaction energies, layer thickness, density, and internal peptide mobility, can be followed for self‐assembled peptide morphologies.^[^ ^136^ ^]^ The structural stability of electrode materials at elevated temperatures is desirable for commercial applications. Classical MD simulations were performed using moles (N), volume (V), and temperature (T) (NVT) and moles (N), pressure (P), and temperature (T) (NPT) ensembles and the thermodynamic equilibrium was studied between 300 and 500 K. The increase in the lifetime of the hydrogen‐bonded networks with successive increase in temperature and its impact on the mobility of these molecules as per Einstein's diffusion coefficient was the primary focus of these studies. It was documented that the hydrogen bonding network was achieved by stacking the peptides into a β‐sheet orientation. Additionally, these membranes also exhibited pores which could facilitate the interaction with electrolyte ions. This study also concluded that due to the high number of hydrogen bonds, thermal stability of the assembled state is retained to a large extent even at 500 K, thereby suggesting that peptide structures are potentially efficient and suitable for organic coatings even for applications where SCs would be used in higher‐temperature ranges.

### 7.2 Peptide/Carbon Systems

Focusing further on the morphology of peptidic systems that promoted good electrochemical performance, Han et al. reported the formation of nanowires of diphenylalanine assisted by reduced graphene.^[^ ^149^ ^]^ The core/shell nanowires were designed by adding the peptide solution in 1,1,1,3,3,3‐hexafluoro‐2‐propanol to the aqueous solution of graphene oxide under mechanical agitation. The formation of the core/shell assembly was more pronounced in the pH range of 3.7–5.4, as observed from the morphological characterizations, which indicated the formation of such assemblies with the help of electrostatic interactions between the positively charged peptide nanowires and the negatively charged graphene sheets. The *I–V* characteristics of the material were studied to evaluate its electrical conductivity. The plot was Ohmic in nature and the resistance was measured to be 2.4 × 10^5 ^Ω with a high electrochemical stability up to a voltage range of 20 V. The cyclic voltammograms of the SC using 0.1 m H_2_SO_4_ as the electrolyte exhibited redox peaks at 0.34 and 0.24 V with a high specific capacitance of 157 F g^−1^. When compared to the planar sheet structures, higher capacitances were identified for the core/shell structures, which demonstrated the obvious effect of morphology on the electrochemical properties.

In attempts to increase the specific capacitance of peptide‐based SCs, Guo et al. developed a SC electrode by doping nitrogen into porous carbon matrices.^[^ ^150^ ^]^ The introduction of heterogenous moieties resulted in larger specific capacitances due to the combined effect of pseudocapacitance as well as EDLC. To enable this, carbonization of biomolecules naturally rich in nitrogen content could be considered as a viable alternative. This would ensure homogenous incorporation of heteroatoms in the carbon matrix of interest. In this configuration, carbon derived from hair was used as a cost‐effective nitrogen‐rich electrode material. Four samples of activated carbon were synthesized and a fifth sample of an activated carbon derived from coconut shell was used as a reference. The obtained activated carbon exhibited a layer‐like morphology with sizes in the microrange and lower crystallinity. X‐ray photoelectron spectroscopy (XPS) analysis illustrated the nitrogen content for all the samples and the exact contributions from the pyridine, pyridinic, quaternary, and oxidized nitrogen contents. The surface area of the activated carbon species was high which indicated its porous nature. High specific capacitances and reversible capacitive characteristics were obtained from the triangular charge–discharge curves. A maximum capacitance of 154.5 F g^−1^ was also recorded with a loss of only 5% after 10 000 cycles at 5 A g^−1^. The pseudocapacitive contributions from the pyrrolic and pyridinic nitrogen were instrumental in assisting the ET from the edges of the graphitic structure and through the quaternary and the oxidized nitrogen moieties, which resulted in high cycling stability and enhanced electrochemical performances.

**Figure 7:**
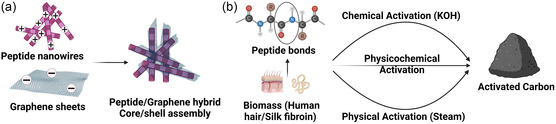
Schematic overview of the methodologies used to prepare peptide/carbon systems. a) Formation of peptide/graphene hybrid core/shell nanowires as a result of electrostatic interactions between the peptide nanowire and reduced graphene oxide. b) Preparation of nitrogen‐rich activated carbon utilizing the nitrogen atoms of the peptide bonds in human hair and silk fibroins through various activation techniques. Figure created with Biorender.com.

Additionally, nitrogen‐rich activated carbons derived from the peptide bonds of silk fibers have also been used as an electrode material for SC applications by Kim et al.^[^ ^151^ ^]^ The activation was performed using steam and KOH to obtain four different types of activated carbon electrodes. The adsorption isotherms indicated greater selectivity of KOH for the silk carbons as indicated by the absence of the hysteresis loop. Consequently, the mesopores were found to be larger for the steam activated carbons. The presence of heteroatoms governs the final capacitance values by contributing in terms of pseudocapacitive behavior. The chemical composition of these heteroatoms in the prepared activated carbons was obtained from the Brunauer–Emmett–Teller (BET) analysis and the pore size distribution curves were obtained using DFT calculations. The pore sizes play an important role in regulating the capacitance of a material. The smaller‐sized pores help in storing capacitive charges whereas the larger pores aid in increasing the mobility of ions. At higher temperatures, greater number of micropores are obtained. The applicability of the prepared activated carbon has been assessed based on two factors, their cost effectiveness and their capacitances. The KOH activated carbons showed almost double the specific capacitance when compared to the steam‐activated silk carbon. However, the values of capacitances are much greater for the activated carbons obtained from both the methods than that for phenolic resin‐based activated carbons standard, thereby aiming at providing a more cost‐effective alternative for the activation of silk carbons. Nitrogen‐doped carbons are significant not just from the perspective of novel capacitive materials with enhanced pseudocapacitive nature, but also because they have been used as substitutes to noble metal/carbon systems as catalysts.^[^ ^152^ ^]^ The high specific capacitances of the derived activated carbons in this work^[^ ^151^ ^]^ coupled with its electro‐oxidative stability promise to introduce a viable method for the preparation of interesting electrode materials for EDLC‐based SCs. The methodologies adopted for the synthesis of these peptide/carbon systems have been illustrated below (**Figure** **7**).

### 7.3 Peptide/Metal Systems

The formation of hybrid organic/inorganic structures which are structurally flexible and ecofriendly is emphasized greatly due to their multifunctional capabilities in the field of energy storage devices. Various transition metal oxides have been used as electrode materials due to their variable oxidation states, distinctive morphologies, large surface area, and high theoretical capacitance. Variable oxidation states make them susceptible to intercalation/deintercalation of electrolyte ions, facilitating fast redox reactions resulting in enhanced pseudocapacitance. One of the approaches which has been adopted by Hu et al. is where the peptide Fmoc‐EF‐NH_2_ was coated with a TiO_2_ layer to solve the problem of dissolution of the peptide in the electrolyte solution.^[^ ^153^ ^]^ The dipeptide sequence developed in this work exhibited a perfect balance between hydrophilicity and hydrophobicity, which is an important aspect to enable sufficient wettability of the electrode surfaces with the electrolyte. The dipeptide system was also capable of forming hydrogels assisted by the aromatic π‐π interactions between the Fmoc groups. TiO_2_ on the other hand exists in various polymorphs such as anatase, brookite, rutile, and TiO_2_ (B). It has been used extensively as electrode materials due to its low cost, practical abundance, and ecofriendly nature. In order to obtain thin uniform films, the peptides were coated on silicon plates and a stacking of the sheets was observed. The device was designed by drop casting the peptide hydrogel on gold‐coated polyethylene terephthalate (PET) surfaces, and polyvinyl alcohol (PVA)/H_3_PO_4_ was used as the electrolyte as well as the separator. The peptide showed capacitive behavior due to its fiber‐like morphology and maintained a rectangular shape even at higher scan rates in its cyclic voltammograms. The absence of redox peaks reiterated the formation of electric double layers which were responsible for the charge transfer. The cycling stability of the peptide‐based device was however seen to diminish to 50 % of its initial values due to its dissolution in the electrolyte. To counter this issue, a layer of TiO_2_ was coated on the surface of the peptide using ALD, which has been reported previously to improve capacitances.^[^ ^154^, ^155^, ^156^ ^]^ This layer not only protected the peptide from dissolution, but it also aided in maintaining its structure even at high temperatures, which is detrimental to the 3D network of the peptide morphology. The modified peptide‐based SC device had a good capacitance retention of 76% even at high scan rates. The conservation of the peptide morphology even after coating with TiO_2_ greatly assisted in reaching the improved electrochemical behavior of the device. The amorphous nature of TiO_2_ was determinant in reaching effective adsorption on the surface of the peptides without disrupting their supramolecular structure. Thus, through the effective combination of a peptide and a metal oxide, it was possible to achieve a green system that could be applied in the future as part of implantable devices.

Self‐assembled thin films of peptide/metal hybrids through electrodeposition have been used to design unique nanostructures. Several organic molecules such as dyes and surfactants have been used in the past for the electrochemical deposition of metal hydroxides. Das et al. synthesized a series of peptide/metal hydroxide nanostructures using electrodeposition for SC applications.^[^ ^121^ ^]^ Three metal hydroxides, Co(OH)_2_, Ni(OH)_2_, and Zn(OH)_2_ and three peptides, Nm‐YW, Nm‐FW, and Nm‐FY (where Nm is naphthalene‐2‐methoxy carbonyl), have been used to prepare nine peptide/metal hydroxide hybrids through electrodeposition. The dipeptides formed from different combinations of tyrosine, phenylalanine, and tryptophan have been capped on the N terminus by naphthalene‐2‐methoxy carbonyl to introduce π‐π interactions which can contribute to the assembly formation. The carboxyl functional group aids in forming bonds with the metal hydroxide to impart structural stability and the amide bonds help in the coassembly between the inorganic and the organic counterparts through intermolecular hydrogen bonding. Indium tin oxide (ITO) was used as the substrate for the deposition of these hybrid films. The morphology of the peptide/Co(OH)_2_ composite had a sponge‐like nature, whereas that of the peptide/Ni(OH)_2_ was nonporous and discontinuous. Peptide/Zn(OH)_2_ had a nanosheet‐like morphology. The effective formation of the composite was confirmed using various characterization techniques. Energy‐dispersive X‐ray analysis (EDAX) confirmed the presence of metal in the films and corresponding metal hydroxide peaks were observed from the Fourier transform infrared (FTIR) spectra. From the diffraction peaks, hexagonal phases of Ni(OH)_2_ and Zn(OH)_2_ were identified. Co(OH)_2_ crystallized following a β polymorphic phase. The CV curves for peptide/Co(OH)_2_ composite in 4M KOH showed little contribution from the peptide. Redox peaks were obtained in the voltammogram, which were characteristic for the redox processes of cobalt hydroxide. The specific capacitance for this system was found to be 3070 F g^−1^. High specific capacitance can be correlated to the porous nature of the material. For the peptide/Ni(OH)_2,_ the specific capacitance is found to be low due to its nonporous structure and for the peptide/Zn(OH)_2_; no redox peak was observed in the CV. The electrochemical efficacy of the composites was found to be poor and elusive; however, the authors presented electrodeposition as a functional approach for the formation of such nanoarchitectures in this study. The role of peptides as structure‐directing agents has also been emphasized.

In a similar study, Singh et al. reported a one‐step in situ electrodeposition of benzo[2,1,3]selenadiazole (BSe)‐protected dityrosine peptide and cobalt hydroxide (BSe‐YY/Co(OH)_2_) on carbon paper as an efficient electrode material.^[^ ^122^ ^]^ For the electrodeposition, cobalt nitrate hexahydrate and the dipeptide were dissolved in a DMSO:water (1:1) solution. Carbon paper was used as the working electrode and the deposition was performed at a potential of −0.9 V for 20 min at 65 °C. The morphology of the nanohybrid was described to be flower like, giving rise to a large surface area and sufficient pores, which are beneficial in initiating improved contact with the electrolyte ions during charge/discharge cycles. BET isotherms revealed its mesoporous nature with a specific surface area of 6.699 m^2^ validating the presence of multiple electroactive sites. The structure of the peptide backbone is influential here in promoting the formation of a stable composite through the secondary interactions between the carboxylate and the metal ions. These interactions were characterized by FTIR through the disappearance of the peak of the carboxylic group for the peptide/metal composite. Additionally, the amide and the aromatic moieties also contribute to the overall structural stability through π‐π stacking and hydrogen bonding interactions. The flower‐like morphology obtained can be visualized as the rearrangement of the sheet‐like morphologies of the peptide/metal composite through these secondary interactions. The electrochemical properties were investigated in 1 m KOH and in 1 M LiOH in a three‐electrode setup. The cyclic voltammograms showed distinct redox behavior due to the reactions arising from cobalt hydroxide as represented in Equation (8) and (9).

(8) $${\text{NO}}_{3}^{-}+H_{2}O+2e^{-}\to 2{\text{OH}}^{-}+{\text{NO}}_{2}^{-}$$

(9) $${\text{Co}}^{2+}+2{\text{OH}}^{-}\to \text{Co}{\left(\text{OH}\right)}_{2}$$

**Figure 8:**
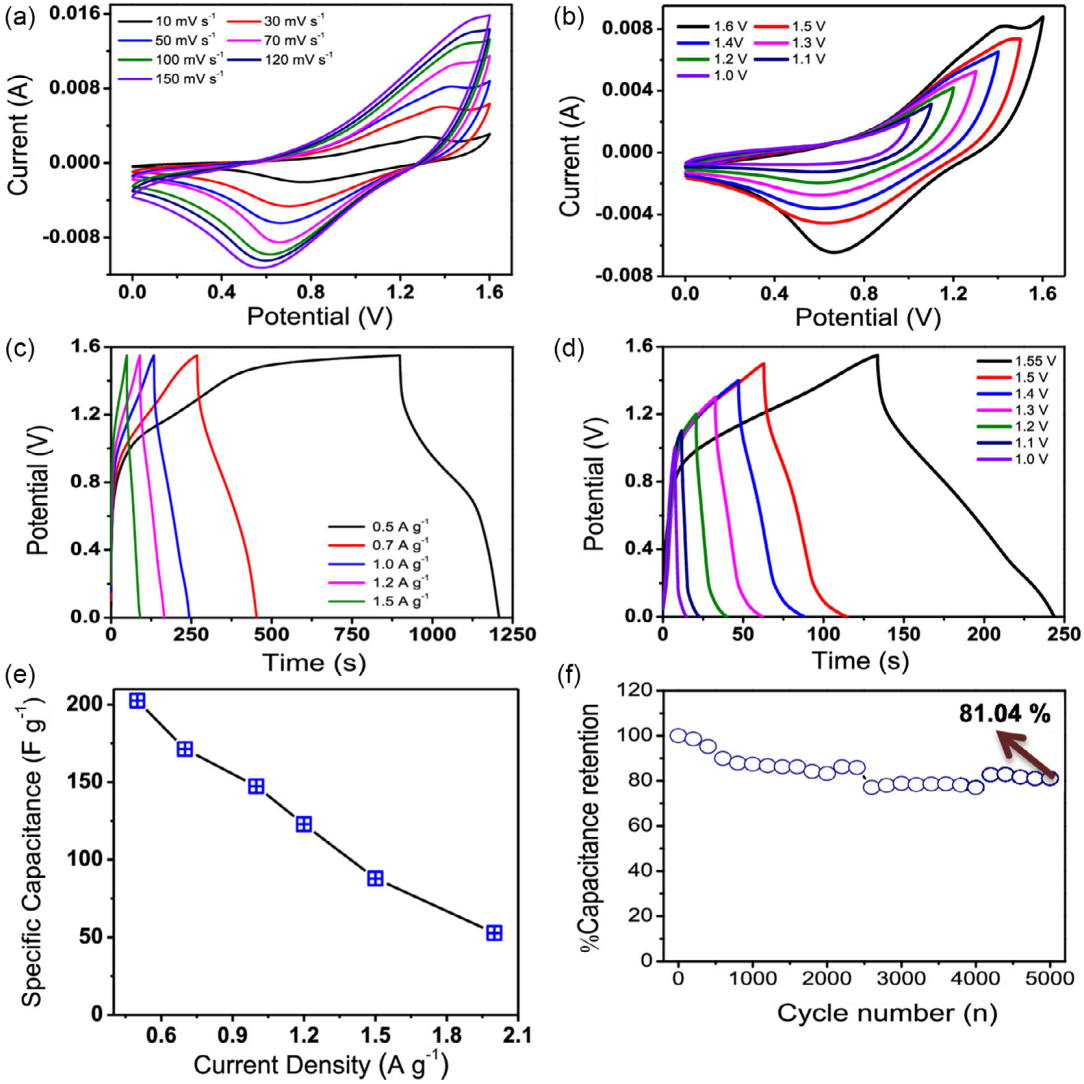
a) CV of the BSeYY/Co(OH)_2_/CP‐based symmetric SC obtained at various scan rates within the potential window of 0.0–1.6 V. b) CV curves for BSeYY/Co(OH)_2_/CP measured at different potential windows at a 50 mV s^−1^ scan rate. c) GCD profiles of the symmetric SC device at different current densities. d) GCD profiles of the symmetric SC device with the upper‐voltage limit values ranging from 1.0 to 1.55 V at a fixed current density of 1 A g^−1^. e) *C*_s_ vs current density plot of the symmetric capacitor device at various current densities. f) Cyclic stability of the symmetric SC device at a constant current density of 2 A g^−1^ for 5000 cycles. a–f) Reproduced with permission.^[^ ^122^ ^]^ Copyright 2021, American Chemical Society.

The galvanostatic charge–discharge curves (GCD) curves are observed to be slightly deviant from the profile of a pure EDLC material. The patterns are indicative of charge transfer and redox behavior arising from the metal hydroxide. A specific capacitance of 974.78 Fg^−1^ at the current density of 1 A g^−1^ was obtained for the nanohybrid. The active surface area of the electrode material was investigated based on the EDLC at the non‐Faradaic regions. The electrochemical active surface area (ECSA) was found to be 8.22 cm^2^, which reiterated the large surface area of the electrode. The impedance spectra displayed a semicircle and a spike at higher and lower frequencies respectively. The charge transfer resistance, *R*_ct_ was lower in 1 m KOH than in 1 m LiOH. This was attributed to the better ionic mobility in KOH due to the larger size of the hydration radius for lithium when compared to potassium. Based on these properties in a three‐electrode setup, a SC device was assembled using the nanohybrid electrodes of the peptide/metal composite deposited on carbon paper and a Whatman filter paper as the separator. PVA/KOH was used as the gel electrolyte. A high energy density of 16.35 Wh kg^−1^ at 0.5 A g^−1^ and a power density of 617.37 W kg^−1^ at 2 A g^−1^ was obtained for the device. The device exhibited a high capacitance retention of 81.04 % even after 5000 cycles and could light up an light emitting diode (LED) when placed in series (**Figure** **8**).

Peptide assemblies have also been used as templates for the directed growth of nanomaterials.^[^ ^157^, ^158^, ^159^, ^160^ ^]^ Green synthesis of nanoparticles has been greatly investigated as an ecofriendly approach to reduce the use of toxic solvents.^[^ ^161^, ^162^, ^163^ ^]^ Peptides are significant in this regard as they have a variety of functional groups which can interact with metal ions and act as reducing and capping agents to promote the formation of metal nanoparticles.^[^ ^164^ ^]^ A short amphiphilic peptide Ac‐I_3_K‐NH_2_ with a hydrophobic tail comprising isoleucine and a hydrophilic head consisting of lysine was synthesized and used as a template for the growth of MnO_2_ nanowires by Du et al.^[^ ^165^ ^]^ The peptide self‐assembled in water to form nanofibers and MnO_2_ nanomaterials were synthesized using varying ratios of KMnO_4_ and Mn(NO_3_)_2_ with and without the presence of the peptide. MnO_2_ was prepared by the usual comproportionation reaction as given in Equation (10).

(10) $$2{\text{MnO}}_{4}^{-}+\;3{\text{Mn}}^{2+}+\;2H_{2}O\to \;5{\text{MnO}}_{2}+\;4H^{+}$$

In the absence of peptide, MnO_2_ formed an aggregation of nanoflakes to form an urchin‐like sphere. In the presence of the peptide, a more controlled deposition of MnO_2_ nanoflakes was achieved on the surface of the peptide nanowire. As the concentration of the precursor was increased, the TEM images showed uniform coating of MnO_2_ nanoflakes around the peptide nanowires. However, at high concentrations, the morphology shifted back to urchin‐like spheres, indicating saturation of the templating capability of the nanowires. The templating ability arises from the electrostatic attractions and secondary interactions between the positively charged side chains of lysine and MnO_4_^−^. The difference in morphology in the presence and absence of the peptide is a function of the distinct nucleation and growth mechanisms. Variable sites are available along the peptide nanowire for the anchoring of MnO_4_^−^ for the subsequent growth of multiple tiny nuclei dispersed all over the nanowire. Orientation and deposition of MnO_2_ nanoflakes take place over it to form a layer of coating on Ac‐I_3_K‐NH_2_. In the absence of the template, there is no scope for the ordered growth of several small nuclei and thus, it assembles into aggregated spheres. An optimum concentration of 15 mM was chosen wherein a uniform coating of MnO_2_ was evident on the peptide nanowire and these peptide/MnO_2_ hybrid nanowires were further characterized and their electrochemical properties were assessed. The effective formation of MnO_2_ was confirmed from the presence of the +4 oxidation state of manganese in the XPS spectrum. Low crystallinity was inferred from the selected‐area electron diffraction patterns and the X‐ray diffraction (XRD) analysis suggested that the peptide nanofibers had little effect on the crystallinity of the synthesized MnO_2_. A high surface area of 275 m^2^ g^−1^ was obtained for the peptide/MnO_2_ hybrid nanowires from the BET experiments along with an average pore diameter of 5 nm. The deposition of MnO_2_ nanoflakes can be visualized as a sequential growth from an individual nucleus, leading to the formation of stacked nanostructures. These stacks enabled the presence of slit‐like mesopores and more active sites for the facile transport of ions. In the case of urchin‐like MnO_2_, the packing of the nanoflakes is more intense, resulting in a decreased specific surface area and narrower pore size. The electrochemical performances of these hybrid nanowires were evaluated based on the cyclic voltammograms and the GCD curves. The CV curves were indicative of pseudocapacitive nature with the presence of small humps. The peak current was linearly dependent on the square root of scan rate indicative of a diffusion‐controlled process. The specific capacitances calculated from the cyclic voltammograms were directly dependent on the morphology of MnO_2_. The highest specific capacitance of 421 F g^−1^ was obtained for the nanowire with the more uniform coating of MnO_2_. Interestingly, almost identical specific capacitances were observed for the nanoparticle without the peptide and the peptide nanowire with a very high (saturated) concentration of MnO_2_. This is due to the comparable morphology in both cases. However, the specific capacitance is slightly higher for the latter due to the more ordered assembly of the nanoflakes when compared to the former. The GCD curves were symmetric in nature and a capacitance retention of 63.2% was achieved for the current densities of 1–5 A g^−1^. The variation in the current densities did not affect the capacitance of the material. Moreover, cycling at 5 mV s^−1^ for 2500 cycles also yielded a high capacitance retention of 93%. All these factors point toward a superior cycling stability. Such a high cycling stability can be attributed to the formation of the core–shell‐type structure. The peptide template offers a strong framework for the development of an interconnected network of nanoflakes, which provides enhanced structural stability that can withstand repeated cycling. Additionally, these types of structures have been reported to possess ample redox active sites, which accelerate contact between the electrode and the electrolyte.^[^ ^166^ ^]^

A similar coassembly of a redox‐active tyrosine‐rich α‐helical peptide template (YYACAYY, Tyr‐C7) in the presence of Ag ions resulted in the formation of a layer‐by‐layer assembly of silver nanosheets.^[^ ^167^ ^]^ Long‐range ET was afforded through the coordination of the Ag ions with the Cys residues between individual peptide monomers. The peptide sequence played an important role in the ET process. The Cys residues selectively coordinated with the Ag ions and on replacing the cysteine with other residues such as glycine, histidine, methionine, or tryptophan, nanosheets were not observed. After the reduction of Ag ions by cysteine, ET is mediated with the help of the tyrosine residues. In the absence of the alanine residues, very few nanosheets were formed. Thus, the synergistic effect of the three residues gives rise to the ET between the nanosheets. The bridging of the Ag ions between the peptide layers was confirmed using Raman spectroscopy and XPS. Multilayered nanosheets were visualized from the TEM and AFM images. To evaluate the ET efficiency, the *I–V* characteristics of the peptide‐templated Ag nanosheets were compared with the commercially available Ag nanowires. Following the conductivity measurements, six tyrosine‐based peptide derivatives (YYAWAYY, YYAMAYY, YYAGAYY, YYAHAYY, Tyr‐C5, and Tyr‐C7) were assembled into solid‐state SC devices using the polymer electrolyte PVA/H_3_PO_4_ and the highest capacitance of 350 μA cm^−2^ at a scan rate of 25 mV s^−1^ was recorded for the Tyr‐C7 peptide. Upon the formation of 2D nanosheets with Ag ions using Tyr‐C7 as the template, the capacitance increased, even further indicating superior ET characteristics.

Carrying forward the onus of designing nontoxic organic SC devices, Tiwari et al. reported the synthesis of a short peptide composed of two phenylalanine (F) units, which were coupled with ferrocene (Fc) and linked to a nucleoside and peptide nucleic acid (PNA).^[^ ^168^ ^]^ Utilizing the spontaneous self‐assembly due to base pairing in the nucleoside domain, also known as the Watson–Crick specific base pairing, guanidine (G) containing PNA dimers could form ordered assemblies with fluorescent properties which could be used as potential materials for organic light‐emitting diodes.^[^ ^169^ ^]^ PNA N‐capped guanidine (G)‐monomer, *N*‐(*N*‐Fmoc‐2‐aminoethyl)‐*N*‐[(*N*‐6‐Bhoc‐9‐guanyl)‐acetyl]‐glycine [Fmoc‐G^NHBhoc^‐*aeg*‐OH], also self‐assembled to form uniform nanospheres.^[^ ^170^ ^]^ Based on these results, nucleopeptide conjugates of FF and PNA G‐monomers using different linkers such as triazole (tz) were explored for their electrochemical properties.^[^ ^169^ ^]^ Following the established semiconducting nature of these peptides, ferrocene‐conjugated peptides were synthesized and their charge‐storage properties were analyzed in this work.^[^ ^168^ ^]^ The synthesis was conducted by click chemistry techniques followed by subsequent coupling reactions. The peptide assembled into a nanotubular structure but upon conjugation with ferrocene, the morphology changed to nanospheres. Interestingly, the attachment of ferrocene on the N‐terminus or the C‐terminus did not have an actual effect on the morphology, and the formation of nanospheres was observed in both cases from the AFM and the SEM images. Enhanced energy storage efficacy was observed for these materials due to their porous morphology. Further on, upon the addition of ferrocene, the promotion of electrochemical characteristics could also be detected. The effects of each of the components on the overall electrochemical properties were studied by utilizing four different systems. FF was functionalized with propyne and conjugated with ferrocene at the N‐terminus to form Fc‐FF‐propyne. It was followed by the addition of nucleoside azide and the azide of PNA to obtain Fc‐Phe‐Phe‐tz‐A^N(Boc)2^ and Fc‐FF‐tz‐aeg‐A^(Boc)2^‐OEt respectively. Finally, triazole‐linked ferrocene was coupled at the C‐terminus to obtain Boc‐FF‐tz‐Fc and at both the N‐ and C‐terminals to obtain Fc‐FF‐tz‐Fc. The specific capacitance was highest when ferrocenes were conjugated to both terminals. The lowering of capacitance values for other systems was attributed to the change in morphology and the obtained capacitances were contributed by the synergistic effects of both the ferrocene and propyne. The cycling stability and the impedance tests were not reported for these systems, but the cyclic voltammograms confirmed the future applicability of such ferrocene‐based peptidic systems as SCs.

Wearable electronics is another important sector which relies heavily on the development of biocompatible materials. SCs have been used extensively in wearable technologies but they suffer from lower energy densities than batteries. This issue can be addressed by coupling them with other energy storage devices. Xiong et al. coupled a peptide–Co_9_S_8_ SC with a triboelectric nanogenerator (TENG) to develop a wearable system.^[^ ^126^ ^]^ Peptides with their intrinsic biocompatibility are excellent candidates for implantable and wearable devices, however, their applicability is restricted due to their instability in highly acidic or alkaline electrolytes. Additionally, they have low power and energy densities, and the peptide bonds are sensitive to higher temperatures. ALD has been used as the technique in this study to coat the peptides on substrates without affecting their morphology significantly. Furthermore, a layer of Co_9_S_8_ has been coated on the peptide layer using ALD to shield the peptide from the effect of the alkaline or acidic electrolyte. Co_9_S_8_ is conducting in nature and therefore it also contributed to the overall capacitance of the device. Ac‐CAAAS_5_ which assembled into nanobricks was used as the cyclic pentapeptide in this study. The peptide exhibited an α‐helical structure as determined by the amide I, amide II, and amide III bands in the FTIR spectrum. Crystalline nature of the peptide was affirmed by the diffraction peaks in the XRD spectra of the peptide. The electrochemical properties of the peptide alone were evaluated by coating it on a bare glassy carbon electrode using 1 m KOH as the electrolyte. Polypyrrole, polyaniline, and carbon nanotubes were used as standards for the comparison of the electrochemical performance of the peptide nanobricks. The cyclic voltammograms indicated that the energy storage characteristics of the peptide were higher than that for conventional conducting polymers, for example, polypyrrole and polyaniline, and it was slightly lower than that for carbon nanotubes. These results were further reaffirmed using electrochemical impedance spectroscopy (EIS), which revealed a low series resistance of 7.4 Ω for the peptide, which is lower than the value obtained for the conducting polymers, and it is comparable to that obtained for carbon nanotubes. These outcomes can be linked to the presence of more active sites in the peptide arising from the nanobrick morphology and improved hydrophilicity which aids the decrease of the interfacial resistance between the electrode and the electrolyte. However, the cycling stability was low for the peptide alone with a capacitance retention of 67% only, which makes it unfavorable for use as electrode materials in SCs. The low cycling stability is due to the hydrolysis of the peptide bonds in the highly alkaline medium. The coating of Co_9_S_8_ was beneficial here as it contributed in imparting higher stability to the system. The SEM images showed clearly that the morphology was maintained even after coating. The presence of the Co_9_S_8_ coating was confirmed using XPS and TEM. The electrochemical performance was investigated after the coating and redox peaks were observed due to the contribution from Co_9_S_8_. Additionally, the capacitance increased by 50 times and 96% of the capacitance was retained even after 500 charge–discharge cycles. These results were further confirmed by assembling a device using peptide‐Co_9_S_8_ as the cathode and activated carbon as the anode. The device exhibited a high energy density of 79 Wh kg^−1^ at a high power density of 1.6 kW kg^−1^. The leakage current was found to be only 9.1 μA and the device retained 98% of its initial specific capacitance even after 50 000 cycles. Its practical applicability was demonstrated by lighting up an LED using these devices in series. Furthermore, the authors devised a self‐powered wearable system using the peptide‐Co_9_S_8_//AC SC device coupled with a TENG. This was an innovative approach toward the development of real‐time devices for commercial applications. The TENG was responsible for converting mechanical energy to electricity, which in turn could be stored by the SC. The TENG/SC system could effectively power an LED for 21 min after being charged for 2.7 h with almost negligible self‐discharging. Furthermore, this system was incorporated into a watch wherein it could detect human pulse signals and convert it into electricity, thereby powering the watch. This integrated system could be considered a great feat in the field of wearable electronics powered by greener technologies.

**Figure 9:**
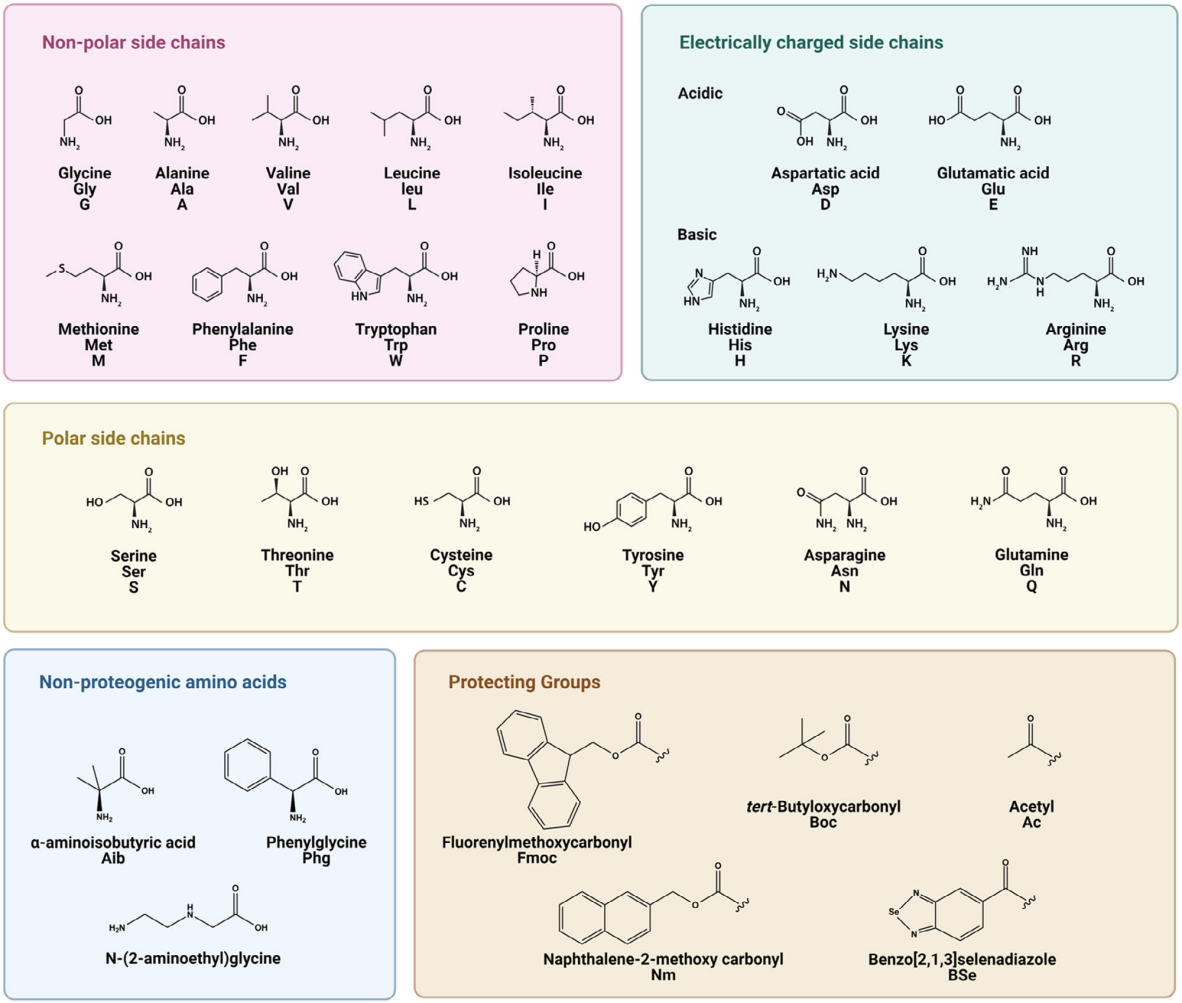
Schematic overview of the most common amino acids, nomenclature, and protecting groups used in the studies covered by this review. Figure created with Biorender.com.

The structures and the nomenclature of the common amino acids as well as the terminal protecting groups are presented in **Figure** **9**; for a quick overview, we also highlight the main aspects of these systems in **Table** **1**.

**Table 1:** Summary of the various peptide‐based systems used for supercapacitor applications

Electron conduction mechanisms, coating, and characterization techniques for peptide‐based systems							
ET mechanisms	Tunneling	Hopping	Superexchange	Flickering resonance	ETp		
Ref	[54–57]	[58–62]	[63,64]	[65]	[30,72]		
Coating techniques	PVD	Electrodeposition	ALD	Other coating techniques			
Ref	[110–112]	[113–122]	[123–126]	[127–130]			
Theoretical models to analyze ET	QM	Combined QM/MM	Statistics‐based FEP, QM/MM‐FEP	Techniques for studying biomolecules in energy storage devices			
Ref	[66–68]	[69,70]	[71]	[131–137]			
Selected complex studies on peptide‐based systems for supercapacitor applications							
Peptide sequence	Morphology	Preparation methods	Device setup		Characterization (Material and electrochemical)	Specific capacitances	Ref
			Electrode	Electrolyte			
Phe‐Phe (FF)	Nanotubes	PVD	Porous Carbon electrodes	1 m H_2_SO_4_	SEM	80 F g^−1^	[111]
					HRTEM		
					Contact angle measurements		
					CV		
Phe‐Phe (FF)	Nanotubes	PVD	Carbon electrodes	0.05 m KH_2_PO_4_/0.5 m KCl	SEM	240 μF cm^−2^	[112]
					CV		
Ac‐CAAAS_5_(X)‐NH_2_	Nanobelts or nanotubes	Drop casting	Glassy carbon electrode	0.1 m H_2_SO_4_	SEM	2.35 mF cm^−2^	[146]
					HRTEM		
				0.05 m KH_2_PO_4_/0.5 m KCl			
X – methyl/phenyl/naphthyl							
					XRD		
					AFM		
					CD		
					FTIR		
					Raman spectroscopy		
					Fluorescence spectroscopy		
					CV		
					GCD		
EFL_4_FE and RFL_4_FR	Nanosheet	–	–	–	MD simulations	18.7 μF cm^−2^	[137]
AAAAAAR	Peptide nanomembrane	–	–	–	MD simulations		[136]
Peptide/Carbon Systems							
Phe‐Phe (FF)	Nanowire	Drop casting	Fluorine‐doped tin oxide (FTO) substrate	0.1 m H_2_SO_4_	SEM	157 F g^−1^	[149]
					TEM		
					HRTEM		
					XRD		
					AFM		
					UV–vis		
					FTIR		
					BET		
					Zeta potential		
					CV		
Nitrogen‐enriched hair‐derived carbons	Noncrystalline layered structure	Slurry (PTFE + Activate Carbon+Acetylene black) coating	Nickel foam	6 m KOH	SEM	154.5 F g^−1^	[150]
					TEM		
					XPS		
					XRD		
					BET		
					CV		
					GCD		
*Bombyx mori* silk fibroin	–	Slurry (PTFE + Active Carbon +Carbon black) coating	Samples pressed into disk shapes as electrodes, glassy carbon as the current collector and glass paper as separator	1 m of tetraethylammonium tetrafluoroborate, (C_2_H_5_)_4_NBF_4_, mixed with propylene carbonate (PC)	TEM	–	[151]
					TGA		
					XPS		
					BET		
					CV		
					Discharge Profile		
Peptide/Metal Systems							
Fmoc‐EF‐NH_2_	Fiber‐like reticular structure	ALD	PET	PVA/H_3_PO_4_	FTIR	125 F g^−1^	[153]
					SEM		
					HR‐TEM		
					CD		
					XRD		
					Raman spectroscopy		
					CV		
					GCD		
Nm‐YW, Nm‐FW and Nm‐FY	Sponge‐like, nanosheets	Electrodeposition	ITO	4 m KOH	FTIR	3070 F g^−1^	[121]
					XRD		
					SEM		
					EDAX		
					CV		
					GCD		
BSe‐YY	Flower‐like	Electrodeposition	Carbon fiber paper	1 m KOH	FTIR	974.78 F g^−1^	[122]
					Raman spectroscopy		
					XPS		
					SEM		
					TEM		
					XRD		
					CV		
					GCD		
					ECSA		
					EIS		
Ac‐I_3_K‐NH_2_	Nanofibers	Slurry (PTFE + Sample+Acetylene black) coating	Nickel foam	1 m Na_2_SO_4_	XRD	421 F g^−1^	[165]
					TEM		
					XPS		
					BET		
					FTIR		
					CV		
					GCD		
YYACAYY	Nanosheet	Drop casting	Stainless steel	PVA/H_3_PO_4_	XRD	350 μA cm^−2^	[167]
					Raman spectroscopy		
					XPS		
					SEM		
					TEM		
					AFM		
					CV		
Fc‐Phe‐Phe‐tz‐Fc	Needle‐like, nanorod or spherical	Drop casting	Toray Carbon electrode	0.1 m potassium phosphate buffer electrolytes at pH = 7.4 and 0.1 m KCl	SEM	2213 μF cm^−2^	[168]
					AFM		
					DLS		
					EDAX		
					CV		
Ac‐CAAAS_5_ (phenyl)‐NH_2_	Nanobricks	ALD	Glassy carbon electrode	1 m KOH	FTIR	1800 F g^−1^	[126]
					XRD		
					SEM		
					XPS		
					HR‐TEM		
					CV		
					GCD		

## 8 Practical Outlook

Even though a steady progress can be observed in the field of peptide‐based SCs due to increased research attention toward the inclusion of more biocompatible materials in various fields, there are certain practical aspects that still need to be considered and addressed appropriately before they can replace traditional systems. Some of these aspects are discussed below.

### 8.1 Cost of Peptides

Since peptide‐based SC technologies are still under conceptualization and are in the developmental stage, the industrial costs are almost impossible to be accurately estimated. Thus, with the increased research interest on the usage of peptidic systems for the development of energy storage devices, it is crucial to briefly analyze the overall cost of large‐scale production of such systems. Peptides could be either isolated from natural sources or they could be chemically synthesized. In most cases, the process of isolation is more laborious and could result in low yields. Alternatively, chemical synthesis of peptides on a large‐scale is usually facile and results in peptides with high purity while matching both commercial needs and profit expectations. This is efficiently exploited in, for example, the pharmaceutical industry, where by now over 80 peptide drugs are approved for human use.^[^ ^171^ ^]^ However, for peptides with longer amino acid sequences, this process could be often self‐limiting and expensive.^[^ ^172^ ^]^ With respect to scale‐up, Gaglione et al. reported the approximate production costs involved in moving from laboratory scale to industrial scale production.^[^ ^173^ ^]^ Interestingly, on increasing the amount of peptides synthesized per batch from 200 to 1000 mg, there is a sharp decrease in peptide cost from 136 to 42 € mg^−1^ with the cost of labor and chemicals being the only two determinants which contributed majorly to the overall cost. These results could be further supported by a similar study by Wang et al. which compared the pharmaceutical applications of therapeutic peptides, biologics, and small molecules.^[^ ^171^ ^]^ In comparison to biologics, therapeutic peptides showed lower production cost coupled with higher membrane permeability, enhanced specificity, and lower immunogenicity. Therefore, based on these observations, we could anticipate a decrease in cost of production for devices using such systems as we move to large‐scale commercial applications. In this respect, other biomaterials such as proteins or DNA could also pose a varied set of problems, such as high cost of the precursor used and the inability to make innovative morphological templates akin to peptides, which could directly impact electrochemical properties. In several cases, expensive metals like platinum and gold have been coated to fabricate e‐textiles^[^ ^174^, ^175^ ^]^ which could also lead to a radical increase in the cost of production. Although peptide membranes have been reported to surpass the capacitance offered by gold‐based SCs,^[^ ^137^ ^]^ the former is still being optimized with respect to their coating efficiencies, environmental sensitivity, and long‐term stability. Therefore, based on these factors, it can be deduced that peptidic systems with minimal toxic effects can serve as a potential alternative to commonly used materials in this regard, as they are relatively cost‐effective and could still work efficiently in the typical physiological environments which biological implants and biomedical devices commonly face.

### 8.2 Foreign‐Body Response

One of the main objectives behind developing biocompatible materials for energy storage devices lies in their potential use as biomedical implants. The major impediment toward realizing this goal is the resistance of the human body toward these artificial materials, giving rise to inflammations around the site of the implant, often referred to as the FBR. The FBRs caused by implanted biomaterials have been reviewed earlier.^[^ ^176^, ^177^, ^178^, ^179^, ^180^ ^]^ Polypeptides have been considered to be efficient anti‐FBR materials and have therefore been used widely in biosensors, drug delivery, tissue engineering, gene delivery, bioimaging, and antimicrobials.^[^ ^181^ ^]^ Functionalizing or coating the surface of implants with AMPs have been used as a facile approach to avoid bacterial proliferation.^[^ ^182^ ^]^ For example, peptide‐coated cardiovascular stent tested in a rat carotid artery model displayed reduced thrombosis and platelet formation, resulting in a 30% reduction in restenosis, which is a major reason limiting the long‐term use of cardiovascular stents.^[^ ^177^ ^]^ Silk protein has also been used in combination with alginate to design a pancreatic islet, which exhibited negligible fibrous tissue formation even after 8 weeks of implantation in a rat model. Similarly, scaffolds developed using silk fibroins, a short peptide which is an alternative splicing product of total insulin‐like growth factor‐1, and electrospun polycaprolactone inhibited FBR responses even after 28 weeks post implantation in a rat subcutaneous implantation model and prevented tissue adhesion in murine adhesion models.^[^ ^183^ ^]^ Additionally, polypeptides with zwitterionic nature displayed improved pharmacokinetics with no histological changes in rats even after three months of administration.^[^ ^184^, ^185^ ^]^ Early host immune responses for a class of multidomain peptide hydrogels were also tested and minimal inflammatory responses were observed for negatively charged peptides.^[^ ^186^ ^]^ Even though, polypeptides have not been reported to elicit FBRs, this could also be due to the fact that natural peptides are easily degraded enzymatically in the body and therefore any long‐term FBRs are difficult to detect. To address this aspect, non‐natural peptides such as β‐peptides which are more stable to enzymatic degradation, have also been tested in this regard. Inspired by the diverse biological applicability of silk proteins, self‐assembled monolayers of poly‐*β*‐homoserine were used as implants.^[^ ^187^ ^]^ These implants resisted FBRs on implantation throughout the duration of 3 months and demonstrated very low collagen capsulation when compared to commonly used PEG hydrogels. Through MD simulations, it was established that the antifouling effect and low FBR activity were attributed to the effect termed as “dual‐hydrogen bonding hydration,” where both the amide groups of the backbone and the hydroxyl groups in the side chain underwent hydration. The anti‐FBR of peptides coupled with the fact that 114 peptides have been approved for theranostic applications till date^[^ ^188^ ^]^ insinuates a promising future of peptide‐based materials for biomedical implants. Therefore, by establishing long‐term effects of these implants through systematic and thorough investigation followed by appropriate clinical trials, they can be developed as next‐generation materials for biomedical applications.

### 8.3 System Considerations

Apart from the cost of developing such systems and the FBRs generated upon using these materials, the appropriate device specifications required for these systems also need to be analyzed for realizing optimal device performances. Most of these specifications are similar to those for conventional SC devices; however, with respect to peptide‐based systems, we discuss few aspects in detail.

For practical applications, the smooth functioning of a SC device is dependent on the ability of the electrode materials to withstand the operating temperatures and the heat generated during the charge/discharge processes. Although Alves et al. theoretically proved that peptide structures can be used even at higher temperature ranges,^[^ ^136^ ^]^ in an actual experimental setup, the stability of peptidic structures in these ranges could prove to be a challenge. However, since peptide‐based materials have been developed as SCs mostly for use at the biotic–abiotic interface such as in implants, biosensors, and other biomedical devices, the efficient functioning of these materials at these temperatures is sufficient to consider them as potential materials for these applications. Additionally, the operating temperature used could also influence the stability of the electrolyte used.^[^ ^189^ ^]^ Therefore, the choice of appropriate electrolytes based on factors such as the type, size, and concentration of the ion in the electrolyte, the interactions at the electrode/electrolyte interface, and the electrochemical potential window contribute significantly to the obtained specific capacitances, the capacitance retention, as well as the energy and power densities.^[^ ^190^ ^]^ Electrolytes can be broadly classified into liquid or solid/quasisolid based on their physical state. In general, liquid electrolytes could be aqueous, organic, or ionic liquids, whereas solid‐state electrolytes could be gel polymer electrolytes or inorganic electrolytes. Each of these types of electrolytes has their distinct set of shortcomings and therefore selecting compatible electrolytes is crucial for the development of novel SC electrodes. For instance, aqueous electrolytes are easy to handle, enable high conductivity and specific capacitance but provide only a short potential window, up to 1.2 V, and have a low energy density and cycling stability.^[^ ^191^ ^]^ Organic electrolytes and ionic liquids offer a higher operational voltage but possess much lower ionic conductivity. Gel polymer electrolytes provide good ionic conductivity but suffer from low mechanical strength and are limited in their operative temperatures due to the presence of water.

With regard to peptide‐based SCs, most of the works reviewed in Section 7 indicate the usage of an aqueous electrolyte with some exceptions (Table 1). For PNT‐based SCs, Beker et al. reported that the double‐layer capacitances increased drastically on changing the electrolyte from KCl to H_2_SO_4_.^[^ ^111^ ^]^ Aqueous electrolytes promoted charge transportation through the nanochannels of the PNT, thereby contributing to the formation of an electrical double layer. The greater mobility of the protons of the acidic electrolyte enabled efficient insertion into the walls of the PNT, resulting in enhanced capacitances. For carbon‐based electrodes, an inorganic electrolyte wets the surface only partially due to the hydrophobic nature of carbon. For PNT, this problem was alleviated and they showed comparable capacitive values in an inorganic solution of 0.05 m KH_2_PO_4_ and 0.5 m KCl in a subsequent study.^[^ ^112^ ^]^ A similar increase in capacitance on changing the electrolyte from 0.05 m KH_2_PO_4_ and 0.5 m KCl to 0.1 m H_2_SO_4_ was also observed in the case of cyclized helical peptides.^[^ ^146^ ^]^ Higher ionic conductivity of the acidic electrolytes enabled efficient electrolyte accessibility for the peptide/graphene core–shell nanowires, resulting in a higher specific capacitance for these systems.^[^ ^149^ ^]^ The contribution of the electrode/electrolyte interaction to the electrochemical performance was studied by Guo et al. where nitrogen‐doped activated carbon derived from peptides of hair showed an increase in capacitance values as a function of the available nitrogen content on the surface of the carbon.^[^ ^150^ ^]^ Organic electrolytes such as 1 m of tetraethylammonium tetrafluoroborate, (C_2_H_5_)_4_NBF_4_, mixed with propylene carbonate (PC) have also been used in this regard for nitrogen‐enriched activated carbons derived from silk fibroins.^[^ ^151^ ^]^ The advantages observed in this case were a higher potential window of 4 V and the larger size of the solvated electrolyte ion as a result of using the organic solvent, PC. As the pore size of the electrode material is closely related to the ion diameter of the electrolyte,^[^ ^192^ ^]^ a positive contribution to the capacitance was obtained due to these factors. Gel polymer electrolyte, PVA/H_3_PO_4_, has also been used to assemble a peptide‐based solid‐state SC device.^[^ ^153^ ^]^ The initial tests on aqueous electrolyte, 1 m H_3_PO_4_, resulted in dissolution of the peptide in the electrolyte solution. Using a layer of TiO_2_ to protect the peptide layer and assembling a solid‐state device using a gel polymer electrolyte, a higher electrochemical performance was achieved. Similarly, Co_9_S_8_ was used as a protective layer on the peptide to preserve it from the harsh alkaline electrolyte, 1 m KOH, in wearable SC devices fabricated by Xiong et al.^[^ ^126^ ^]^ For peptide/metal hydroxide hybrid nanostructures, the high porosity of the electrode material arising from their sponge‐like morphology results in electrochemical accessibility of the OH^−^ ions of the alkaline 4 m KOH electrolyte enabling fast Faradic redox reaction, leading to high specific capacitances.^[^ ^121^ ^]^ Electrodeposited peptide/cobalt hydroxide films were also tested in the presence of 1 m KOH and 1 m LiOH as electrolytes.^[^ ^122^ ^]^ The effects of the hydrated ionic radius of the Li^+^ and K^+^ cations were evaluated by tracking the electrochemical performances. Higher capacitance and lower solution and charge transfer resistance were observed on using KOH as the electrolyte. This was attributed to the fact that the hydrated ionic radius of the Li^+^ ions is greater than that of the K^+^ ions, which limits ionic mobility and disrupts interactions at the electrode/electrolyte interface. To formulate the SC device for practical applications, PVA/KOH was used as the electrolyte which resulted in the highest capacitance due to the higher ionic conductivity of the gel polymer electrolyte. Furthermore, for pseudocapacitive materials, the electrolytes contribute majorly to pseudocapacitance by facilitating redox reactions between the electroactive materials and the ions of the electrolyte. For the peptide‐templated synthesized MnO_2_ hybrid nanowires, the charge‐storage mechanism proceeded through an ion‐coupled redox reaction which involved the surface adsorption of the Na^+^ cations from the Na_2_SO_4_ electrolyte on MnO_2,_ thereby contributing to the overall pseudocapacitance.^[^ ^165^ ^]^ Additionally, in view of developing materials that can be used in biocompatible devices, 0.1 m potassium phosphate buffer electrolytes at the physiological pH = 7.4 and 0.1 m KCl were used for investigating the electrochemical properties of diphenylalanine and ferrocene conjugates.^[^ ^168^ ^]^ Retention of the redox‐active nature of the ferrocene in the conjugate coupled with enhanced electrochemical performance was observed for these peptides. Apart from these experimental approaches, a green amino acid ionic liquid was also used as electrolyte to assemble SC device using a theoretical approach by Colherinhas et al.^[^ ^137^ ^]^ The capacitance values observed in this case were higher even when compared to gold electrodes simulated in the same setup. Therefore, a wide range of electrolytes are applicable for the peptide‐based systems, that grant diverse combinations and room for optimization to specific needs. Moreover, most of the reported systems use conventional coating techniques for the preparation of electrode materials such as ALD, PVD and drop casting. Both ALD and PVD are reliable in this respect for the development of thin and uniform films for these applications. Specialized PVD setups for coating peptide‐based systems have also been reported by Rosenman et al. which have been shown to produce high capacitances.^[^ ^110^ ^]^ In addition to this, other techniques need to be evaluated for different classes of peptides before a generalized setup can be recognized for future applications.

## 9 Conclusion

Peptides with their synthetic flexibility and biocompatibility have been used as prototypes to mimic and understand various endogenous processes. These attributes also make them susceptible to forming self‐assemblies and coassemblies with ease. By altering the length and nature of the amino acid sequences and the side chains, these materials can be easily tailored to obtain desired application performance. This aspect was particularly explored to develop SCs utilizing the supramolecular structures. However, the formation of these assemblies is subject to various extrinsic and intrinsic factors, and therefore, reproducing similar architectures on a large scale to achieve electrochemical properties matching previous fabrications could prove to be a challenge. Nonetheless, the unique morphologies obtained in these cases were the major contributing factors in obtaining high specific capacitances. Earlier reports focused mostly on dipeptide FF as a model system for SCs. The nanotubular morphology contributed significantly to the electrochemical properties and exhibited capacitances higher than carbon nanotubes. However, this approach could be deemed as a substantial step toward the fabrication of greener alternatives to traditionally used carbon nanotubes, the high mechanical and thermal stability of the latter is difficult to achieve. The investigation into other self‐assembling peptide systems leads to the use of cyclized helical peptides which showed higher specific capacitance than other reported peptide‐based SCs. Theoretical methods were also employed to analyze octapeptides which exhibited a high theoretical specific capacitance. However, reproducing these results in an experimental setup would require considerations on additional parameters, rendering this direction nontrivial. Further on, hybridization or composite formation has been promoted as an effective technique in this regard. Peptide/metal and peptide/carbon systems were developed using the polar residues in peptides, which aided in forming covalent bonds and enabled highly efficient electron transport kinetics. The porous structures and the unique morphologies obtained in these cases played key roles in enhancing the specific capacitances due to increased surface areas, improved wettability, and the availability of more electroactive sites. Peptides also proved to be good structure‐directing agents and were used as templates and surfactants to grow nanoparticles. Nonetheless, the scope of these systems was mostly confined to a few examples and an elaborate and systematic set of studies using various metal oxides/hydroxides and carbonaceous materials is lacking. Another potential drawback we need to mention is the dissolution of peptides in electrolyte solution. To counter this problem, different coating techniques were used to deposit the peptide films on electrode surfaces and an additional layer of electroactive material was deposited on top of it to guard the peptide film from dissolution. This approach could however increase the cost of production of such devices. Also, coating techniques should be highly optimized to obtain good adhesion to solid substrates, and the formation of uniform films needs to be ensured to obtain enhanced specific capacitances.

In overall, the high capacitance observed in numerous studies, the applicability of a wide range of already established coating techniques and electrolytes, and the significantly decreasing unit costs exemplified by large‐scale pharmaceutical production all point toward efficient future use of peptide‐based SCs. Further benefits involve the fact that peptides are anti‐FBR materials, which is supported by numerous pharmacological and theranostic examples and by long‐term studies on murine models. It can be concluded that these peptidic systems have the potential to become one of the commonly used set of molecules in energy storage solutions for future biomedical implants or externally wearable electronics operating at a biotic/abiotic interface. Although the challenges above need to be overcome, nevertheless, by now the numerous listed studies support that exciting new directions could be tackled with applying supramolecular peptide constructs for SCs. These devices could clearly lead to suitable future prototypes of next‐generation high‐performance biocompatible and biodegradable energy storage systems.

## Conflict of Interest

The authors declare no conflict of interest.

## Author Contributions

The manuscript was written through the contributions of all the authors. The final version has been approved by all the authors.
